# Open ventilator evaluation framework: A synthesized database of regulatory requirements and technical standards for emergency use ventilators from Australia, Canada, UK, and US

**DOI:** 10.1016/j.ohx.2022.e00260

**Published:** 2022-01-07

**Authors:** Kate Kazlovich, Soumya Ranjan Mishra, Kamran Behdinan, Aviv Gladman, Jesse May, Azad Mashari

**Affiliations:** a Institute of Biomaterials and Biomedical Engineering, University of Toronto, Toronto, ON, Canada; b Department of Mechanical and Industrial Engineering, University of Toronto, Toronto, ON, Canada; c Mackenzie Richmond Hill Hospital, Toronto, ON, Canada; d Department of Anesthesiology and Pain Medicine, University of Toronto, ON, Canada; e Department of Anesthesiology and Pain Management, Toronto General Hospital, University Health Network, Toronto, ON, Canada

**Keywords:** COVID-19 medical equipment, Emergency ventilators, Pandemic ventilator, Open-source medical hardware, Design standards, Rapid manufacturing, Electromechanical devices, Open-source hardware, Performance evaluation, Risk analysis

## Abstract

Development of emergency use ventilators has attracted significant attention and resources during the COVID-19 pandemic. To facilitate mass collaboration and accelerate progress, many groups have adopted open-source development models, inspired by the long history of open-source development in software. According to the Open-source Hardware Association (OSHWA), Open-source Hardware (OSH) is a term for tangible artifacts — machines, devices, or other physical things — whose design has been released to the public in such a way that anyone can make, modify, and use them. One major obstacle to translating the growing body of work on open-source ventilators into clinical practice is compliance with regulations and conformance with mandated technical standards for effective performance and device safety. This is exacerbated by the inherent complexity of the regulatory process, which is tailored to traditional centralized development models, as well as the rapid changes and alternative pathways that have emerged during the pandemic. As a step in addressing this challenge, this paper provides developers, evaluators, and potential users of emergency ventilators with the first iteration of a pragmatic, open-source assessment framework that incorporates existing regulatory guidelines from Australia, Canada, UK and USA. We also provide an example evaluation for one open-source emergency ventilator design. The evaluation process has been divided into three levels: 1. Adequacy of open-source project documentation; 2. Clinical performance requirements, and 3. Conformance with technical standards.

**Specifications table:** 

Hardware name	Framework for review and evaluation of emergency use ventilation systems
Subject area	• Educational tools and open-source alternatives to existing infrastructure • Clinical Engineering • Emergency Medicine • COVID-19 medical equipment
Hardware type	• Mechanical ventilation systems (medical application)
Closest commercial analog	Commercially manufactured and distributed mechanical ventilation systems used to support or completely replace spontaneous breathing of the patient.
Open-source license	Creative Commons Attribution-ShareAlike 4.0 International license (http://creativecommons.org/licenses/by-sa/4.0)
Cost of hardware	N/A
Source file repository	https://doi.org/10.17632/xcm62gpxvk.1

## Hardware in context

Innovation in medicine requires a constant balance of potential benefits and risks. The history of medicine down to the present holds many examples of harmful innovations [1], [2], [3]. Accelerated development and clinical deployment of novel devices during a global crisis, with its attendant stresses on health care systems, presents additional and distinct challenges. First, securing of essential human and material resources for rapid development and manufacturing of urgently needed devices in the face of limited timelines, and supply chain and infrastructure disruptions; second, assuring the level of effectiveness and safety required of a minimally viable product while ensuring that the device does more good than harm to patients and health care workers.

The COVID-19 pandemic has re-invigorated a movement that emerged during the 2003 SARS epidemic to address critical shortages of ventilators. Ventilators present a striking case study of the balancing of benefits and hazards, given the urgency, the technical and physiologic complexity, and the severity of potential harms they entail.

Confronted with rapidly escalating demand, established ventilator manufacturers have sought to increase their production capacity and in some cases support manufacturers in other sectors to retool for ventilator manufacturing. Medtronic has released the design of the Puritan Bennett™ 560 ventilator under a temporary permissive license (set to end by the official resolution of the pandemic or 2024). This has led to effective public–private partnerships such as Ventilators for Canadians [4] producing regulatory approved ventilators based on designs with well-established track records. As of mid-November 2020, this group had delivered nearly 6000 devices. Despite these successes, the complexity of established commercial ventilators has prevented rapid scaling at the rates necessary to meet the demands of peak infection periods. In addition, the fate of these devices after the expiry of the permissive license is uncertain and it is likely that the devices will be decommissioned. Contracts awarded by the US federal goverment since 2010, specifically for the development of streamlined, low-cost ventilators for pandemic preparedness, resulted in devices that reached the final stages of pre-market clearance at the Food and Drug Adminstration before being ultimately abandoned by the companies involved. These contracts did not include requirements for public disclosure of resulting designs and associated data, and the life-saving work has not been made available for pandemic manufacturing [52].

Other ventilator initiatives have set out to develop devices with minimum viable functionality which could be manufactured rapidly, and at a reasonable cost. Working largely without the backing of established medical device manufacturers and their development teams, these groups have sought to address the resource challenge by adopting open-source intellectual property models to leverage mass collaboration in the development and manufacturing processes. The open-source approach is further motivated by the need to ensure that deployment of successful designs is not bottlenecked by the centralization of production that is often associated with traditional intellectual property restrictions. This has been effective in rapidly mobilizing a largely volunteer workforce with access to diverse expertise and resources and has led to a wide array of projects exploring a range of technical approaches with varying degrees of success [5]. The challenge of is that of ensuring an essential level of effectiveness and safety has been the subject of significant efforts by regulatory agencies and their affiliates. Confronted with the challenge of adapting a complex and multi-faceted evaluation process that typically takes 2–5 years to complete [6] and which varies significantly by jurisdiction, some agencies have provided guidance documents and modified standards for emergency use ventilators (EUVs) to support accelerated temporary clearance to market devices while seeking to minimize potential hazards. Health Canada (HC), UK’s Medicines and Healthcare products Regulatory Agency (MHRA), USA Food and Drug Administration (FDA), and Australia’s Therapeutic Goods Administration (TGA) have all released documents to this effect. However, there remains a significant gap in the application of these requirements to the development process of open-source ventilators. This is in large part due to the significant variability, lack of clarity and occasional divergence of the requirements across jurisdictions, which do not reconcile easily with the diverse affiliations of transnational project teams. While these teams often have significant medical and engineering expertise, they frequently lack contributors with a deep understanding of medical device risk management procedures and regulatory processes.

It is important to note that the qualifications and standard operating procedures of traditional medical device manufacturers play a vital role in patient safety through implementation of quality systems that conform to technical standards (such as those by the International Electrotechnical Commission (IEC) and the International Organization for Standardization (ISO)) and faciliate regulatory compliance. Under the current state of emergency however, some requirements for manufacturers have been relaxed and auditing activities and external oversight have been dramatically curtailed.

In this paper we seek to take a first step in providing a review of risk management and regulatory approval processes and synthesizing the available guidance documents into a unified framework and requirements database for the evaluation of EUVs. Such a framework has two primary use cases:

1) It is intended to support ventilator developers in creating project roadmaps and providing formative evaluations throughout the development process that integrate risk management principles and regulatory requirements, thus facilitating the translation of technical innovations into clinical practice.
2) Components of the framework can be applied to systematic evaluation of projects and candidate devices for the purpose of device selection, or identification of patterns of technical deficiencies across the field to guide research investment. The overall approach described here can be universally applied to the development and evaluation of any open-source medical device.

## Medical device product development and regulatory approval process

### The risk management approach

Despite differences in structure and mandate, a central focus of all regulators has been the assessment, evaluation and mitigation of safety risks emanating from medical devices and substances, and ensuring that such risks that remain are outweighed by the likely benefits. For much of the history of modern regulation however, this focus has been implicit in various technical standards. Since the 2007 release of *ISO 14971: Application of Risk Management to Medical Devices* [7], a risk management framework has become central to the regulatory approval process in most jurisdictions.

Now in its third edition, the 36 pages of ISO 14971:2019 [7] and the accompanying guidance document ISO/TR 24971:2020 [8], present a clear and concise conceptual framework for medical device safety. This standard integrates assessment, evaluation, and mitigation of risks into every step of the medical device life cycle, from the initial definition of goals and specifications all the way to clinical deployment, maintenance, decommissioning and disposal. The document does not prescribe specific tools and techniques for risk assessment and control, as these are highly specific to devices, clinical indications and use cases. Rather it consists of high-level process requirements that need integration into all stages of the device life cycle. Unlike most ISO standards, this standard is self-contained, but is referenced by virtually all other key standards related to medical device development.

Not only is this standard endorsed by all device regulatory agencies and central to all approval processes, but it also provides a very pragmatic and clear approach to the device development process that can be applied in all contexts, including the fast-paced development of emergency use devices such as EUVs. In fact, in situations of global disruption when the usual checks and balances of the regulatory system may not be fully operational, the application of this framework by device developers is even more essential to maximize the benefit-risk ratio of novel devices. Below we summarize the standard. Keywords from the standard are italicized. We highly recommend reading the entirety of this short but rich document for all those interested in any aspect of medical device development or evaluation.

Perfect safety is not possible. Any medical device, no matter how carefully designed and tested will contain numerous *hazards*, that is, potential sources of *harm*. These hazards remain latent until *hazardous situations* arise, either during the devices’ *intended use*, or during *reasonably foreseeable misuse*. Such a situation exposes the device user or patient to the hazard that may, in the absence of corrective actions, lead to harm to the patient, care provider, environment or property. Personal harms are not limited to the physical (for example a false cancer diagnosis).

Central to the framework is the benefit-risk ratio. *Benefit* is the positive outcome from application of the device. *Risk* consists of the probably and severity of harm. The *risk assessment process* consists of risk *analysis* – that is the identification of hazards and estimation of their associated risks – and risk *evaluation*, the comparison of the estimated risks against pre-defined criteria for acceptable risk set by the manufacturer. Various approaches for the determination of risk criteria are discussed in the guidance document ISO/TR 24971:2020 [8]. Risks that exceed acceptable levels must be addressed with *risk control* measures to bring the *residual risks* within acceptable bounds. In order of priority, risk control measures are categorized as: 1) design modifications to the device or manufacturing process that effectively eliminate the hazard; 2) addition of features or manufacturing process modifications to reduce the risk; and 3) provision of information, alteration of use cases or user training to address the risk. This process is iterative, with risk control measures being in turn subject to risk analysis to identify any new hazards that might arise from them. While some components of the above processes can be quantified and subjected to probabilistic modeling, the process has inherent uncertainties, and the standard does not prioritize quantitative over qualitative methods for addressing these.

Safety is not an isolated property of the medical device. Hazards and their associated risk profiles are a function of complex interactions between the device, the patient, the indication for use, the operators, and the surrounding environment. As in other critical systems, human error is a major cause of medical harm [9]. Device usability and human factors evaluations are therefore a major component of risk analysis and control interventions. Considerations of device usability, ergonomics and human factors are especially significant to patient and provider safety during large-scale emergencies, when health care workers are under additional stress, may be working outside of their regular roles, and without adequate supports from engineers and technical staff. During the current pandemic, the shortage of ventilators has received far greater attention than the equally critical shortages of staff adequately trained to safely operate, maintain, and monitor them. An unsafe life-saving device is an ineffective life-saving device. It is therefore imperative that major contributors to device usability such as interfaces, connectors, labels and markings, user manuals and reference documentation be as well designed and efficient as possible. The primary technical standards for medical device usability are the collateral standards IEC 60601-1-6 and 1-8 [10], [11], and IEC 62366:2015 [12].

The risk management process is to be applied to the entire device life cycle, from development to disposal. Devices that perform to specifications once deployed but require unmanageably complex or costly maintenance or release unacceptable levels of toxins and persistant waster during disposal would not meet the requirements of the standard. Given the uncertainties involved in the assessment process, a key component of the risk management consists of post-deployment monitoring of device use and safety events, which may alter prior risk estimates and require reassessment and implementation of additional control measures. While the various consequences of a global public health emergency may curtail an adequate assessment of life cycle risks beyond the development stage, some considerations such as device durability, shelf life, storage and required maintenance need to be considered even under the most urgent circumstances.

### The regulatory approval processes

Regulatory approval of medical devices consists of meeting regulatory requirements and technical standards related to not only the specific device but also the manufacturing facility and processes. Process requirements including quality management, administration, record keeping, sales and distribution practices, are defined by specific standards that certified medical manufacturers must meet to produce Class II or higher devices. Most jurisdictions require registration and periodic audits of manufacturing establishments, and adherence to Good Manufacturing Practice standards [13]. In most cases, manufacturers must be certified under *ISO 13485:2016 Medical Devices – Quality Management Systems – Requirements for Regulatory Purposes* [14]. The evaluation framework discussed here is unfortunately limited to requirements and standards that apply to the device itself and does not include requirements and standards that apply to the manufacturing process.

Regulatory approval processes vary significantly across jurisdictions both in their mandate and implementation. The United States, Canada, Australia, and Japan have centralized, public sector regulators (such as the FDA) whereas the European Union system is largely decentralized [15], [16], with specific requirements and approvals issued by private, for-profit companies known as “Notified Bodies” which are certified by governmental agencies known as “National Competent Authorities” such as the UK’s MHRA [17]. Kramer et al. [15] and Van Norman [6] provide a cross-national comparison and more detailed overview of the US FDA process, respectively.

Despite differences in implementation, there is significant family resemblance among the processes based on the risk management approach. Most rely on a 3-level classification of devices by potential risk. Where class I represents the lowest risk category (e.g., toothbrush, stethoscope) and class III the highest (e.g., implanted devices). The assessment of device risk is based on several factors, such as: the intended use, life cycle, degree of invasiveness, similarity to previously approved devices and whether it incorporates a medicinal substance. The approval pathway increases in complexity and rigor with increasing risk class. According to the US FDA, European Commission’s Medical Device Coordination Group (MDCG) and HC, an emergency ventilator is classified as a class II or IIa device [18], [19], [20], [21] that indicates moderate risk.

Class I and II devices are subject to less stringent regulatory requirements than class III. Approvals for the former typically do not require detailed clinical testing. Class II devices such as novel ventilators that are sufficiently similar to already approved devices are often approved based on technical specification and bench-testing results to demonstrate that the device performs its intended function and complies with technical standards for functionality and safety. In the US this information is submitted as part of the pre-market notification submission (PMN, also known as a 510[k]) to the FDA. Clinical evidence of efficacy based on human trial data is typically not required, but reviewers may require formal usability testing and human factors evaluation [2].

In 2004, as part of its efforts to address national emergency preparedness in the aftermath of the 9/11 attacks, the US congress passed the Project Bioshield Act. One of the provisions of the ACT was the creation of the Emergency Use Authorization process for expedited and temporary regulatory approval of devices required to address widespread emergencies. Four conditions are required for an EUA to be issued: 1) presence of a serious or life-threatening condition caused by a known agent; 2) reasonable belief that the product may be effective in prevention, diagnosis, or treatment of the condition; 3) the known and potential benefits of the device outweigh the known and potential risks; 4) there is no adequate, approved, and accessible alternative to the product [22]. Other jurisdictions have comparable expedited approval processes. The focus of these expedited regulatory evaluations is two-fold: first, ensuring that the device performance meets a set of essential clinical parameters, and second, that the device complies with the most critical technical standards to minimize potential hazards.

These requirements place significant constraints on the product development process. However, despite significant efforts by regulatory bodies to facilitate the approval processes, open-source EUV development initiatives, with their transnational developer communities, are confronted with a challenging collection of performance and technical requirements from multiple jurisdictions, which frequently leads to disregard of regulatory requirements until a late stage in the development process. This approach can lead to substantial backtracking and loss of misdirected resources. Given the urgency and resource limitations associated with the development of emergency use devices it is advisable to incorporate required standards and performance parameters into target specifications and project road maps at an early stage.

### Evaluation of ventilator designs and readiness

Ventilators comprise a range of devices designed for various use cases, time courses, and settings. These include treatment of acute respiratory distress syndrome in intensive care units, ventilation of patients under general anesthesia for surgery, short-term ventilation of critically ill patients during transport or off-site diagnostic testing, and long-term ventilation of patients with chronic neuromuscular diseases. This paper focuses specifically on the EUVs. These devices are intended as temporary, last-resort devices for situations where standard, approved devices are not accessible. They are required to provide minimum viable functionality, safety features, and to be optimized for simplicity, reliability, as well as rapid manufacturing.

The proliferation of open-source ventilator designs over the course of the COVID-19 pandemic has led to efforts at collation and classification of the projects to evaluate the openness, technological readiness, and deployment viability. One of the most prominent of these is the work by Pearce [23], which presents an overview of the latest developments and common challenges. The review points to the database assembled by Read and colleagues [5], which tracks all publicly announced emergency ventilator projects and provides a high-level rating of each project in terms of openness, feasibility of manufacturing, community support, functional testing, reliability, COVID-19 suitability, and clinical friendliness[5].

### Open ventilator evaluation framework

Current publicly accessible ventilator databases provide general assessments of performance metrics directed at the validity and suitability of various approaches for further research and investment. There remains a need for a standardized, open evaluation framework that addresses essential design and performance metrics in a manner that is commensurate with existing, multi-lateral regulatory requirements.

Our team sought to develop the first iteration of such an evaluation framework for the design and development of EUVs. This open-source tool consists of a database and handbook that synthesize currently available performance and technical requirements from the regulatory agencies and their associated technical advisory organizations. At the time of this publication, regulatory guidelines and technical guidance documents were available from governmental and affiliated technical agencies in Canada, USA, UK, and Australia. We hope that this initiative will attract participation from developers, regulatory agencies, and health care administrators, to become an increasingly effective tool for facilitating the rapid translation of technical innovations into beneficial advances at the frontlines of patient care.

The technical evaluation component of the framework is applicable to all EUVs regardless of intellectual property licensing. However, given the number of nominally open-source projects in the space we have also included a section to pragmatically evaluate the openness of projects and adequacy of the publicly shared data, based on best practices recommended by the Open-source Hardware Association (OSHWA). There are currently no recognized standards in this regard specific to open-source medical devices.

## Research methodology

The “Open Emergency Ventilator Evaluation Framework” consists of a database of published regulatory requirements with references to specific sections of source documents and technical standards. An accompanying handbook documents the database.

The database is a synthesis of existing regulatory guidelines on emergency ventilation systems. Currently it is limited to English-language documents. At the time of this undertaking, Health Canada, USA FDA, UK MHRA, and Australia’s TGA had produced guidelines or guidance documents specifically addressing emergency use ventilators during the COVID-19 pandemic [21], [19], [24], [25]. These documents were comprehensively reviewed by the authors and discussed in regular panel meetings. References to other regulatory and national or international technical standards were exhaustively traced and collated in a database. Classification systems included in the source documents were preserved, and a synthesized unifying classification system was added by the authors. An outline of the primary source documents and their content is provided below.

### Overview of currently available regulatory guidelines and related standards on emergency ventilators

The regulatory bodies cited in this paper have provided performance requirements of varying levels of detail. MHRA and TGA have also provided checklists and templates. These documents refer to technical standards from the International Organization for Standardization (ISO), International Electrotechnical Commission (IEC) and their affiliated national bodies. In addition to their primary standards, some of these organizations have released documents to facilitate application of standards during the pandemic. These documents were also reviewed, and their relevant content included in the database. In cases where the copyright on the source document precluded quoting of a section, detailed references to the source document were entered into the database instead.

• International Organization for Standardization (ISO): ISO has made available several relevant standards, many of which are currently accessible for read-only access at no charge. Of specific interest is the new standard for emergency medical service environments: “ISO 80601-2-84:2020 Medical electrical equipment – Part 2-84: Particular requirements for the basic safety and essential performance of ventilators for the emergency medical services environment” [26].
• British Standards Institution (BSI): To support COVID-19 response efforts and in collaboration with the MHRA, BSI has also made several standards accessible at no charge. For a complete least, please refer to the BSI website [27].
• American National Standards Institute (ANSI) [28]: Standards related to the pandemic are available at no charge in read-only format.
• Canadian Standards Association (CSA) [29]: Provides a complimentary access to their “COVID-19 Response Standards & Handbooks” available to any emergency ventilator developers.
• Association for the Advancement of Medical Instrumentation (AAMI): AAMI has published a consensus report on the development of Emergency Use Ventilators (EUV) [30]. This document highlights the elements most relevant to EUV development from ISO 80601-2-80:2019 “Medical Electrical Equipment – Part 2-80: Particular Requirements for Basic Safety and Essential Performance of Ventilatory Support Equipment for Ventilatory Insufficiency” [31]. AAMI also provides an End User Disclosure guidance document and a template test report for emergency use ventilators.

### Reconciliation of terminology

Synthesis of the documents required standardization of terminology pertaining to the necessity of specific requirements. For example, the MHRA classifies requirements into: “‘must’ — identifying mandatory minimum viable product requirements; ‘should’ — identifying highly desirable features that enhance therapeutic benefits; and ‘could’ — identifying features that are desirable, but which do not significantly enhance the performance of the system.” [24].

Similarly, the ISO regulations use auxiliary verbs to define levels of mandated requirements. As stated by the ISO: “ ‘shall’ — means that conformance with a requirement or a test is mandatory for conformance with this document; ‘should’ — means that conformance with a requirement or a test is recommended but is not mandatory for conformance with this document; ‘may’ — is used to describe permission (e.g., a permissible way to achieve conformance with a requirement or test); ‘can’ — is used to describe a possibility or capability; and ‘must’ — is used to express an external constraint.” [32].

To synthesize and organize all relevant regulatory information, our team performed an extensive review of standards and checklists that are available to the public and can be accessed or purchased online. All evaluation checklists provided by the reference agencies were reviewed and incorporated into the framework (most notably those by AAMI).

The initial version of the framework presented here includes all requirements that were identified as mandatory by at least one of the referenced regulatory bodies. Desired or recommended features that were not identified as essential to the safety and performance of such system by any of the regulators have also been included but have not been reviewed exhaustively and may be incomplete. Complete inclusion of these secondary requirements is a future goal of the project.

### Database structure

The database is stored in a spreadsheet and can be accessed from the project repository (https://doi.org/10.17632/xcm62gpxvk.1). Specific requirements are divided into categories and levels of evaluation. Subsets of requirements can be sorted and extracted by any of the existing categories; for example, requirements from a single regulatory agency, a specific level of evaluation or a specific domain can be quickly identified.

To increase flexibility and accommodate a range of use cases, the requirements have been classified by several schemes. In addition to the 3-level structure described in this paper, these include the original classifications from the source documents, as well as a synthesized classification system developed by our team. More use-case specific classification schemes can be readily added. Users are thus able to extract subsets of requirements to suit specific needs and create customized checklists. An example application of the evaluation framework is illustrated in the final section.

The “Description” column provides a general description of the regulatory requirement or testing specification. The “Priority” section allows the user to identify or sort by priority level (discussed above) either as determined by a single agency, or a synthesis of all agencies included. The “Standards Reference” section provides citations to all relevant standards and clauses that the user should follow to retrieve complete information. Due to the very strict copyright requirements pertaining to most technical standard documents, we were not able to quote relevant sections directly in most cases. This is a significant usability limitation of the current tool.

## Results

### Framework structure: Levels of evaluation

The framework is structured into three sequential levels, with each successive level analyzing data from previous stages at higher resolution. Level 1 assesses the availability and adequacy of information provided for the design under evaluation. Level 2 assesses basic clinically relevant performance parameters. Level 3 assess compliance with technical standards.

#### Level 1 – Data accuracy and documentation

Level 1 evaluates the adequacy of available open-source data to undertake further evaluation of the design. While the Framework, and this stage specifically, are tailored to open-source projects, it can also be applied to closed-source projects so long as the data specified is accessible to the evaluator. The assessment covers licensing and the completeness of provided technical documentation for independent replication of the device and further development. The assessment checklist used in this section is based on recommendations from the Open-source Hardware Association (OSHWA).

OSHWA provides a checklist [33] that is used as part of its open-source certification process and outlines its standard criteria:

• Documentation that comprehensively describes the components required for building the complete device
• Design files that can be modified and distributed by others, in formats that allow for changes (e.g., native file formats compatible with open-source CAD software; formats that can be read by not easily edited in other software, such as. step files, are not optimal)
• Bill of materials
• Manufacturing and assembly instructions, including instrumentation and explanation of design decisions and revision history
• Licensing documentation
• Software code and documentation
• Hosting of project in publicly accessible repository

As these recommendations are not specifically for medical devices, they do not account for the requirements of regulatory approval and standards conformance. Comprehensive, well-organized, appropriately concise, and readable documentation is a major determinant of success at virtually every stage of the device life cycle. Some of the most significant technologies underlying the growth of open-source software are those that facilitate effective documentation, document organization and version control. During device design, high quality and up-to-date documentation is essential for the effective coordination of large, distributed development teams. Many of the nominally open-source EUV projects have inadequate public documentation to make meaningful open collaboration possible.

The regulatory approval process is also based largely on a review of project documentation. Maintaining efficient and effective project documentation processes is a significant challenge in medical device development, which can be especially acute for open-source projects with their largely decentralized structures. The regulatory guidelines and technical standards included in the OVEF frequently identify required components that must be documented; however, there is no specific standard or authoritative guidance document that explicitly outlines a comprehensive documentation package. High-level requirements for documentation are outlined in ISO 13485:2016 Medical Devices – Quality Management Systems – Requirements for Regulatory Purposes [14]. Most commercial device developers rely on commercial software systems that implement the Quality Management System standards, provide templates, and document management infrastructure for this purpose. There are currently no open-source quality management system tools available for medical device development.

Documentation requirements that were reviewed as part of this project can be roughly broken down into the broad files listed below. Documentation may be in any form, including written, audio or video as appropriate. These areas have significant overlap, and in such cases, a documentation system permitting cross linking and minimizing duplication of documents is recommended. The risk management file for example, may largely consist of an organized set of links to documents from other sections.

• *Device design:* To describe the detailed structure and function of the device, including component specifications, design files, testing performance and regulatory compliance documents.
• *User documentation and training:* Instructions for deployment and use including transportation, assembly and installation, operation, regular maintenance, decontamination and infection control, and trouble shooting. This must include a maintenance manual and outline potential risk mitigation measures that have been implemented via user documentation and training, as outlined in the risk management file. This category also includes documentation on device labelling and markings and their compliance with relevant standards and regulations.
• *Risk Management File:* Complete documentation of processes established for risk management (following ISO 14971:2019), including the results of the risk management process for the device: an adequate list of identified hazards and the methods and sources used to identify them, their associated risks assessment and control measures.
• *Design History:* Includes all original and revised design versions that may include design files, testing procedures, device performance evaluations, updated compliance documentation, user recommendations or any other relevant design changes. This document is especially critical for open-source projects as it provides critical orientation to any developers seeking to contribute to the project.
• *Testing Reports:* Comprehensive test reports covering the all-testing history for the device. Test reports should include protocols, procedures, raw data, analysis, and results. Raw data inclusion should only be restricted by requirements to protect any personal health information for testing involving human subjects. This file should include formative testing performed during the iterative development process as well testing to evaluate conformance with regulatory requirements and technical standards.

Further work is needed to develop specific consensus standards on the documentation for open-source medical hardware.

#### Level 2 – Clinical performance parameters

Level 2 provides guidance for the assessment of minimal performance requirements for the ventilator to provide adequate respiratory support. This level focuses primarily on what the device does (functionality), as opposed to how it does it (mechanism), which is the focus of Level 3. This section is based on documents provided by the national regulatory agencies listed above. It includes minimum viable performance requirements that will satisfy all the regulatory guidelines for emergency use ventilators included in the framework.

The clinical performance parameters were consolidated from a number of regulatory standards such as: AAMI EUV 60601-1, ISO 80601-2-80:2019, and ISO 80601-2-84:2020 [30], [31], [26]. These primary parameters are extensive and provide a detailed description of all performance metrics and testing procedures. The collateral standards can be further explored in Level 3 of the framework. The majority of these parameters are modified to support the design and development efforts directed towards addressing the shortage of medical devices at a time of global pandemic. Thus, several non-essential performance parameters have been eliminated to help streamline the process.

#### Level 3 – Technical standards compliance

Level 3 covers the technical standards to which the design must adhere to provide reasonable assurance of device safety and meet requirements set-out by regulatory agencies. This assessment level evaluates the underlying mechanisms and operator interactions through which the device satisfies the functional requirements covered in Level 2. Most of the key standard references refer to the risk management framework detailed in ISO 14971 [7].

While we do not have the complete checklist to complete Level 3 evaluation, we have made reasonable effort to outline all relevant criteria. In Table 1, we have listed the standards necessary for developers based on 3 categories, namely: General Standards, Particular Standards and Collateral Standards

**Table 1:** A list of general, particular, and collateral standards relevant to level 3 device evaluation.

**Categorization**	**Recommended Standards**
General Standard	IEC 60601-1 (2012)
Particular Standard	ISO 80601-2-80 (2019)
Collateral Standards	ISO 147971 (2019) ISO 10993 (2018) IEC 60601-1-2 (2014) ISO 18652-1 (2017) ISO 18652-2 (2017) ISO 18652-3 (2017) ISO 18652-4 (2017)

It is of utmost importance to note that detailed information of each standard’s requirements cannot be provided due to the copyright policy of the regulatory agencies such as the ISO/IEC, AAMI, ANSI, BSO, etc. these standards were temporarily made available for developers in readable forms on their respective websites during the pandemic. However, the clustered requirements in this paper (mechanical, electrical, software, usability and human factors, risk management process etc.) are derived from the review of several standards.

### Biocompatibility

The increasing use of 3D printing and other additive manufacturing techniques in medical devices, especially during the pandemic, has raised concerns regarding several potential risks. These include quality control, chemical and physical properties of components, contaminants introduced during the printing process, biocompatibility of materials, porosity, and mechanical properties of printed parts. The FDA and other agencies are working to develop standards and processes for assessment of such devices and components. A 2017 report by the FDA [34] outlines the primary concerns and direction of this work.

Biocompatibility of the components in contact with the patient or the respiratory gas stream is a significant and challenging concern for open-source developers. Many groups have addressed this by relying on off-the-shelf standard ventilation components such as circuit tubing, filters, valves, and connectors. With the notable exception of bag-valve-mask based designs however, which can maintain all novel components outside of the respiratory gas stream, most proposed EUV designs involve the use of blowers, adapters, valves, bellows, or other components in the gas path, which have not been previously approved for medical use. Printed components made with materials that are already well established in medical devices —such as acrylonitrile butadiene styrene (ABS) or polyethylene terephthalate (PET) variants— pose far lower risks regarding biocompatibility and contamination of the gas stream provided original materials are of high quality and the printing process well controlled. For more novel materials or complex and critical parts made with 3D printing, a more rigorous evaluation for biocompatibility and mechanical properties may be required. Key standards related to biocompatibility are:

• ISO 10993: Fifth Edition 2018-08: Biological Evaluation of Medical Devices – Part 1: Evaluation and Testing Within a Risk Management Process [35].
• ISO 18562 First Edition 2017-03: Biocompatibility Evaluation of Breathing Gas Pathways in Healthcare Applications, Parts 1–4.
  • Part 1: Evaluation and Testing Within a Risk Management Process [36].
  • Part 2: Tests for Emissions of Particulate Matter [37].
  • Part 3: Tests for Emissions of Volatile Organic Compounds [38].
  • Part 4: Tests for Leachables in Condensate [39].

### Electrical design

The growing pool of modular open-source microcontroller platforms and components such as those developed by Arduino™ [40] have been instrumental in the progress of open-source hardware. Like 3D printers, these electronic hardware and software tools were originally developed for prototyping but are increasingly used in small-scale production. Numerous proposed EUV designs rely on these platforms for most or all electronic and software control functions. While these platforms may satisfy small and medium scale production requirements for some consumer devices, they are not currently able to provide the reliability and robustness required for critical medical devices operating in standard health care enviornments.

Translating early prototypes to production ready designs requires addressing a number of significant hazards related to electrical design. Some examples are robustness of the enclosure against fluid spills and repeated exposure to cleaning and decontamination measures; redundancy of critical components or other mechanism to address the risk of electronic component failure or processing error; reliability of the electronic components for prolonged continuous operation; and contingency mechanisms for loss of electrical power supply to the device. The main relevant standards in this regard are:

• IEC 60601-1: 2012: Medical Electrical Equipment – Part 1: General Requirements for Basic Safety and Essential Performance [41].
• ISO 80601-2-80:2019 Medical Electrical Equipment – Part 2–80: Particular Requirements for Basic Safety and Essential Performance of Ventilatory Support Equipment for Ventilatory Insufficiency [31].
• ISO 80601-2-84:2020 Medical Electrical Equipment – Part 2–84: Particular Requirements for The Basic Safety and Essential Performance of Ventilators for The Emergency Medical Services Environment [26].

ISO 80601 builds upon the general standards (GS) of IEC 60601-1 and provides additional specific requirements for ventilators [26], [31]. AAMI has provided guidance regarding the specific application of ISO 80601-2-80:2019 to EUV devices [31]. MECA has also made available an evaluation package to support compliance with these standards [42], though the latter is not specific to emergency use devices. Invacare Inc. has also made an example of a complete conformance document available on-line [43].

Requirements related to electromagnetic interference (EMI) testing in IEC 60601-1-2 [44] have been waived for EUVs, based in part on the assumption that these devices are unlikely to utilize high-energy electrical systems and generate significant EMI. However, the operation of EUV systems may still create sufficient EMI to interfere with sensitive medical devices in the vicinity. Thus, while specific testing requirements have been waived, consideration of these requirements in the selection of components and design of the device enclosure is highly recommended.

### Software design

Most EUV devices rely on software driven controls. While initially simple, these software components have grown in complexity as projects have advanced. With increasing complexity comes increased risk of software-related device malfunctions. Even comparatively simple devices such as drug infusion pumps have been implicated in deaths related to software errors [45], [46].

The safety of software in medical devices can benefit significantly from open-source development. Numerous resources exist on best practices for the management of software repositories for general and research purposes [47], [48]. Established practices in the open-source software community have led to the development of highly robust and reliable code bases that underlie some of the most critical components of the global digital infrastructure. It is well recognized that under the right circumstances, mission critical code such as cryptographic security systems, benefit significantly from transparency and open-source development models that expose potential errors to many more corrective eyes than closed source development. These practices should form the basis for software development for open-source medical devices in general.

The primary technical standard for medical device software is *IEC 62304: 2015: Medical Device Software – Software Life Cycle Processes* [49]. MHRA [24] currently provides the most specific recommendations in relation to software for EUVs. Most importantly, they include application of a risk management process to software development, as documented by:

1. Software Development plan

2. System and software requirements specifications

3. Appropriate software architecture and software design documents

4. A risk management plan and report

5. Software verification and validation plans and reports

6. A software release note

7. Limitations

## Validation and characterization: open-source ventilator evaluation framework applied to the RepRapable Automated Bag-Valve-Mask Ventilator

In this section, we present a sample evaluation for the RepRapable Automated Bag Valve Mask-based Ventilator — an open-source ventilator developed and manufactured by the Michigan Technological University [50]. None of the authors has a connection to this project. The evaluation was performed independently, based on publicly available data consisting of a journal article [50] and the project repository (https://osf.io/fjdwz/). Project developers were not contacted for additional information. This is not intended to be a comprehensive evaluation of this specific design but an illustration of the proposed evaluation framework in action.

The device is an automated bag valve mask (BVM) compression system, which may serve as a temporary emergency ventilator. It incorporates an Arduino-based controller with a real-time operating system. The device has been designed so that most of its mechanical components can be manufactured using open-source 3D printers based on the pioneering RepRap system.

Level 1: Data Adequacy and Documentation — Summary of Level 1 evaluation is presented in Table 2. This design is shared via a well-structured and maintained repository and satisfies most of the OSHWA criteria. The project documentation also includes bench testing data.

**Table 2:** Level 1 Evaluation: Data adequacy & documentation of the RepRapable ventilator.

Documentation that adequately describes all components of the project's building process from scratch	**Intent of Design:** Clearly Explained **Assembly Instructions:** Clearly Explained **Operating Instructions:** Clearly Explained
Design files that can be modified and distributed by others, in formats that allow for changes (i.e., native file formats compatible with the open-source CAD software)	**Mechanical:** Provided **Electrical:** Provided
Bill of materials	**Mechanical:** Clearly Listed **Electrical:** Clearly Listed **Accessories:** Clearly Listed
Manufacturing and assembly instructions, including instrumentation and explanation of design decisions	**Mechanical:** Clearly Listed Electrical: Clearly **Listed Software:** Clearly Listed
Licensing documentation	**License:** GNU GPL V3
Software code and documentation	**Firmware:** Available **Logic Nuances:** Clearly Explained
Hosting of project in publicly accessible repository	**Test Setup:** Clearly Illustrated and Explained **Standardized Specifications:** Clearly Illustrated and Explained

Level 2: Performance Assessment — Complete Level 2 evaluation checklist is available via Mendeley Data repository. Results related to critical operational parameters are presented in Table 3. The evaluation is based on reported results in the repository. These results only partially satisfy the critical operation parameters. The device failed to attain recommended positive end expiratory pressure (PEEP) levels and several required parameters are not reported. No data on life cycle testing has been provided.

**Table 3:** Level 2 Evaluation: Performance Assessment of the RepRapable ventilator –critical operation parameters.

**Parameters**	**Required Range (MHRA)**	**Achieved Parameters**	**Verdict**
FiO2	21–100%	Not Included	Fail
Vt	50–800 ml	100–846 ml	Pass
RR	10–30 bpm	5–45 bpm	Pass
I:E	1:1–1:4	1:1–1:4	Pass
PEEP	5–20 cmH_2_O	2–11 cmH_2_O	Fail
Peak Inspiratory Pressure	≤PP + 2 cmH_2_O	Not Included	Fail
Plateau Pressure	32–35 cmH_2_O	Not Included	Fail

Level 3: Standards Compliance — Assessment of the RepRapable ventilator design’s detailed standards compliance is presented in Part 2 of this paper, including chapters that discuss relevant assessment metrics and testing protocols associated with hardware, software, electrical, and mechanical systems, as well as risk and human factors assessment pathways.

**Fig 1:**
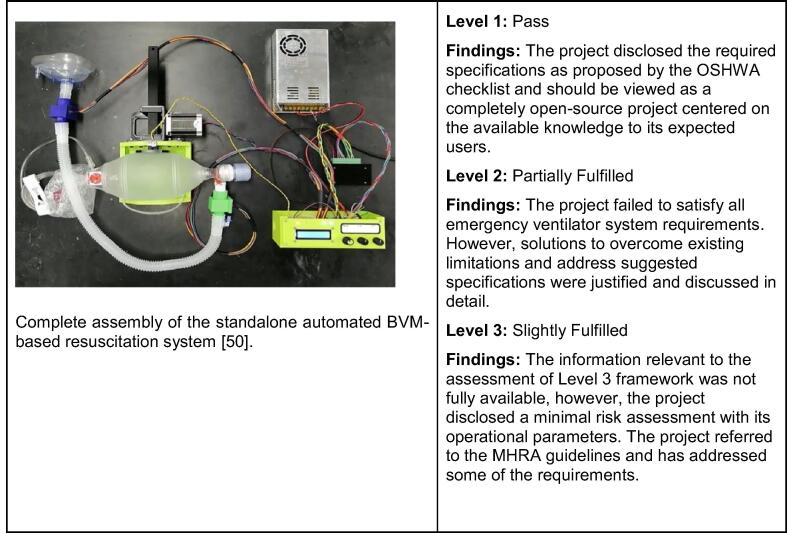
Partially RepRapable automated opensource BVM based ventilator [51].

Fig. 1 provides a short summary of the overall assessment performed with the help of the outlined framework.

## Conclusion

Tracking and complianceying with the intricate web of technical standards poses perhaps the most daunting challenge for the development of regulatory approved open-source class II and III medical devices. This challenge is substantial even for experienced engineers working in established device companies. The application of these standards to specific devices in the development and approval processes has spawned a sizable consulting industry which is generally inaccessible to open-source projects with their financial constraints. Even access to the very standards documents themselves, with their highly stringent copyrights and significant cost can be a significant barrier.

The development of EUVs during the current pandemic has benefited greatly from the support of ISO and other agencies in providing free read access to critical standards, and the provision of guidance documents by regulators and their affiliates such as AAMI [30]. However, it remains the case that the majority of the standards referenced in EUV requirements are not readily accessible and lack associated guidance documents with official sanction. Strick copyright restrictions on international standards, even ones currently available for free viewing, prevents the inclusion or linking of specific content in third party databases or other tools aiming to improve the usability of these standards and facilitate their application. In such cases, we have had to limit the information in the database to references to the specific section of the source. We have endeavored to make these references as specific as possible. Therefore, use of the framework, especially for Level 3 evaluation, must be done in conjunction with the source standards documents.

The requirements included in the Open Ventilator Evaluation Framework are those that apply to the device itself; however, regulatory approval of medical devices also includes evaluation of the administrative organization and manufacturing facilities that will be used to produce and distribute the device. These latter requirements are beyond the scope of this work.

Finally, while we have made every effort to ensure a comprehensive synthesis of the existing regulatory requirements, it is inevitable that the database will include some errors. In addition, given the dynamic nature of pandemics and emergencies, it is likely that the source documents will be modified in the future and supplemented with additional documents and resources. We hope that users of this open resource will in time correct and improve upon this work and support our efforts to maintain an up-to-date and effective tool for translating open-source innovations into safe, effective, and regulatory approved devices for the care of patients.

## CRediT authorship contribution statement

**Kate Kazlovich:** Methodology, Data curation, Investigation, Writing – original draft, Project administration. **Soumya Ranjan Mishra:** Methodology, Data curation, Investigation, Writing – original draft, Validation. **Kamran Behdinan:** Supervision, Resources. **Aviv Gladman:** Supervision. **Jesse May:** Resources, Writing – original draft. **Azad Mashari:** Supervision, Writing – original draft, Conceptualization.

## Declaration of Competing Interest

The authors declare that they have no known competing financial interests or personal relationships that could have appeared to influence the work reported in this paper.

## References

[1] Institute of Medicine, Medical devices and the public’s health: The FDA 510(k) clearance process at 35 years, Med. Devices Public’s Heal. FDA 510(k) Clear. Process 35 Years, pp. (1–318)(2011), doi: 10.17226/13150.

[2] C. Sorenson, M. Drummond, Improving medical device regulation: The United States and Europe in perspective, Milbank Q., 92 (1) (2014), 114–150 doi: 10.1111/1468-0009.12043. 10.1111/1468-0009.12043 PMC3955380 24597558

[3] Maak T.G., Wylie J.D. (2016). Medical device regulation: A comparison of the United States and the European Union. J. Am. Acad. Orthop. Surg..

[4] Ventilators for Canadians (V4C) [Online]. Available: https://ventilatorsforcanadians.com/ (2020) (accessed 11.04.21).

[5] R. Read, COVID-19 Ventilator Projects and Resources and FAQ, [Online]. Available: https://github.com/PubInv/covid19-vent-list. (2020).

[6] G. A. Van Norman, Drugs, devices, and the FDA: Part 2, JACC basic to Transl. Sci., 1 (4) (2016), 277–287, doi: 10.1016/j.jacbts.2016.03.009. 10.1016/j.jacbts.2016.03.009 PMC6113340 30167516

[7] ISO 14971 (2019).

[8] ISO/TR 24971:2020 Medical devices – guidance on the application of ISO 14971 [Online]. Available: https://www.iso.org/standard/74437.html (2020).

[9] Kohn L., Corrigan J., Donaldson M. (1999). To err is human: building a safer health system. Natl. Acad. Press. Inst. Med..

[10] IEC 60601-1-6:2010+AMD1:2013+AMD2: 2020: Medical electrical equipment – Part 1-6: General requirements for basic safety and essential performance – Collateral standard: Usability, Geneva, Switzerland, (2020).

[11] IEC 60601-1-8: 2006: Medical electrical equipment — Part 1-8: General requirements for basic safety and essential performance — Collateral standard: General requirements, tests and guidance for alarm systems in medical electrical equipment and medical electrical systems, Geneva, Switzerland, (2006).

[12] IEC 62366-1: 2015/AMD 1: 2020: Medical devices — Part 1: Application of usability engineering to medical devices — Amendment 1, Geneva, Switzerland (2020).

[13] Food and Drug Administration, General Controls for Medical Devices, pp. (1–4) [Online]. Available: https://www.fda.gov/medical-devices/regulatory-controls/general-controls-medicaldevices. (2014) (accessed 11.23.20).

[14] ISO 13485:2016 (2016).

[15] D.B. Kramer, Y.T. Tan, C. Sato, A.S. Kesselheim, Ensuring medical device effectiveness and safety: a cross--national comparison of approaches to regulation, Food Drug Law J., 69 (1) (2014). PMC4091615 24772683

[16] French-Mowat E., Burnett J. (2012). How are medical devices regulated in the European Union?. J. R. Soc. Med..

[17] C. Sorenson, M. Drummond, Improving medical device regulation: The United States and Europe in perspective, Milbank Q., 92 (1) (2014), 114–150 doi: 10.1111/1468-0009.12043. 10.1111/1468-0009.12043 PMC3955380 24597558

[18] Competent Authorities for Medical Devices. [Online]. Available: https://www.camdeurope.eu/locations/ (accessed 11.23.20).

[19] FDA, Ventilators and Ventilator Accessories for COVID-19, 2020. [Online]. Available: https://www.fda.gov/medical-devices/coronavirus-covid-19-and-medical-devices/ventilatorsand-ventilator-accessories-covid-19. (accessed 11.23.20).

[20] Medical Device Coordination Group Working Groups, European Commission Medical Device Coordination Group (MDCG), 2020. [Online]. Available: https://ec.europa.eu/health/md_dialogue/mdcg_working_groups_es (accessed 11.23.20).

[21] Health Canada, Interim Order respecting clinical trials for medical devices and drugs relating to COVID-19: Notice – Canada.ca, Health Canada, 2020. [Online]. Available: https://www.canada.ca/en/health-canada/services/drugs-health-products/covid19- industry/interim-order-respecting-clinical-trials-medical-devices-drugs/notice-interimorder.html#a2 (accessed 11.23.20).

[22] Beninger P. (2020). COVID-19: regulatory landscape of medicinal and medical device products for human use. Clin. Ther..

[23] Pearce J.M. (2020). A review of open-source ventilators for COVID-19 and future pandemics. F1000Res..

[24] MHRA, Exemptions from Devices regulations during the coronavirus (COVID-19) outbreak, Gov.Uk, (2020) [Online]. Available: https://www.gov.uk/guidance/exemptions-from-devicesregulations-during-the-coronavirus-covid-19-outbreak. (accessed 11.23.20).

[25] Therapeutic Goods Administration, Ventilators and other devices intended for respiratory support for COVID-19, TGA, (2020) [Online]. Available: https://www.tga.gov.au/behind-news/ventilatorsand-other-devices-intended-respiratory-support-covid-19#alternatives. (accessed 11.23.20).

[26] ISO 80601-2-84:2020 (2020).

[27] BSI, COVID-19 Response – Ventilator, 2020. [Online]. Available: https://www.bsigroup.com/enGB/topics/novel-coronavirus-covid-19/ventilators/. (accessed 11.23.20).

[28] ANSI, ANSI – COVID19, 2020.[Online]. Available: https://www.ansi.org/news_publications/news_story?menuid=7&articleid=27ba33a0-7482- 47c5-b3a7-faa8a55518eb. (accessed 11.23.20).

[29] CSA, COVID-19 Response Standards & Handbooks, 2020. [Online]. Available: https://www.csagroup.org/news/covid-19-response-standards-handbooks/. (accessed 11.23.20).

[30] AAMI, AAMI COVID-19, 2020. [Online]. Available: https://www.aami.org/news-resources/covid19-updates/coronavirus-resources-for-the-field. (accessed 11.23.20).

[31] ISO 80601-2-80:2019 (2019).

[32] International Standards Organization, ISO Foreword and Supplementary Information. [Online]. Available: https://www.iso.org/foreword-supplementary-information.html. (accessed 11.23.20).

[33] OSHWA, Open-source Hardware Certification. [Online]. Available: https://certification.oshwa.org/process.html (2020) (accessed 11.23.20).

[34] Food and Drug Administration, Technical Considerations for Additive Manufactured Medical Devices: Guidance for Industry and Food and Drug Administration Staff Document, Cent. Devices Radio l. Heal., pp. (1–30) (2017).

[35] ISO 10993 (2018). Evaluation and Testing Within a Risk Management Process.

[36] ISO 18562-1 (2017). Evaluation and Testing Within a Risk Management Process.

[37] ISO 18562-2 (2017).

[38] ISO 18562-3 (2017).

[39] ISO 18562-4 (2017).

[40] Arduino, Suggested hardware for COVID-19 emergency equipment projects. [Online]. Available: https://www.arduino.cc/covid-19/hardware-support, (2020) (accessed 11.23.20).

[41] IEC 60601-1: 2012: Medical Electrical Equipment – Part 1: General Requirements for Basic Safety and Essential Performance, International Electrotechnical Commission, Geneva, Switzerland (2012)

[42] MECA – Medical Equipment Compliance Associates, IEC 60601-1: Download Free Compliance Documents. [Online]. Available: https://60601-1.com/information/, (2020) (accessed 11.23.20).

[43] IEC 60601-1 Medical Electrical Equipment, PDF, Invacare Inc. [Online]. Available: https://invacaredocs.com/assets/documents/PIM/e513fc6a0b948b84cf0f8ff2c42189a87b6d3bf 7_64.66T.14.168.01__iec60601_1h.pdf, (2020).

[44] IEC 60601-1-2: 2014: Medical Electrical Equipment Part 1-2: General Requirements for Basic Safety and Essential Performance – Collateral Standard. Electromagnetic Disturbances – Requirements and Tests, International Electrotechnical Commission, Geneva, Switzerland (2014).

[45] Deaths Linked to Drug Infusion Pump Overdosing; Software Error Is Root Cause. Journal of Clinical Engineering. 2005 Mar;30(1):12–13. [Online]. Available: https://journals.lww.com/jcejournal/Fulltext/2005/01000/Deaths_Linked_to_Drug_Infusion_Pu mp_Overdosing_.21.aspx.

[46] M. Llamas, Thousands of Infusion Pumps Recalled After Several Injuries and a Death, Drug Watch. [Online]. Available: https://www.drugwatch.com/news/2020/08/03/infusion-pumpsrecalled-after-injuries-and-death/., (2020) (accessed 11.23.20).

[47] Jiménez R.C. (2017). Four simple recommendations to encourage best practices in research software. F1000Res..

[48] Open-source Guides, How to contribute to open source. [Online]. Available: https://opensource.guide/,(2020) (accessed 11.23.20).

[49] IEC 62304: 2015: Medical Device Software – Software Life Cycle Processes, International Electrotechnical Commission, Geneva, Switzerland (2015).

[50] Petsiuk A., Tanikella N.G., Dertinger S., Pringle A., Oberloier S., Pearce J.M. (2020). Partially RepRapable automated open source bag valve mask-based ventilator. HardwareX.

[51] S. Mishra, Emergency Use Ventilator Evaluation and Assessment: Open-Source Hardware, Performance, Regulatory Requirements and Technology Readiness (Master of Applied Science), 2021. University of Toronto. Available: http://hdl.handle.net/1807/104873.

[52] Mar 29, 2020. Kulish N, Kliff S, Silver-Greenberg J. The U.S. Tried to Build a New Fleet of Ventilators. The Mission Failed. The New York Times. (Accessed 14 January 2022).

