# Supplementary material for: Open ventilator evaluation framework: A synthesized database of regulatory requirements and technical standards for emergency use ventilators from Australia, Canada, UK, and US
Source: HardwareX. 2022 Jan 7;11:e00260. doi: 10.1016/j.ohx.2022.e00260 (PMC8752315; doi:10.1016/j.ohx.2022.e00260)
Supplement: Supplementary data 3 [file mmc3.docx]

**Evaluation Guide**

**Table of Content**

**Introduction**

Purpose

Use Case

Considerations

Limitations

1. **Level 1 Evaluation**
   1. Open-Source Definition
   2. Assessing Open-Source Designs
   3. Documentation
      1. *Design Files*
      2. *Bill of Materials*
      3. *Instructions and Explanation*
      4. *Licensing*
      5. *Software*
   4. Hosting Open-Source Project
   5. Buildability Considerations?
2. **Level 2 Evaluation**
   1. Mandatory Parametric Requirements
      1. *FiO2 & O2*
      2. *PEEP*
      3. *Flowrate*
      4. *Tidal Volume*
      5. *Respiratory Rate*
      6. *Inspiratory & Expiratory Ratio*
      7. *Inspiratory Airway Pressure*
   2. Recommended Parametric Requirements
      1. *Inspiratory resistance during the resuscitator expiratory phase*
      2. *Spontaneous breathing with the gas input pressure outside the rated range*
      3. *Respiratory Resistance*
      4. *Resuscitator dead space and dead space of airway accessories*
      5. *Inspiratory flow*
      6. *Threshold pressure for initiation of*
      7. *Peak inspiratory flow*
      8. *Pressure limitation under normal use*
3. **Level 3 Evaluation**
   1. Regulatory Requirements
      1. *Guide Through Health Canada*
      2. *Guide Through MHRA*
      3. *Guide Through FDA*
      4. *Guide through TGA*
   2. Required Standards
      1. *Guide Through ISO and IEC*
      2. *Guide Through AAMI*
      3. *Guide Through MECA Checklist*
4. **MIT Emergency Ventilator** (or other alternative) **Evaluation Sample**
5. **Database Navigation**

**Introduction**

**Purpose:**

The purpose of this document is to serve as a guide towards the assessment and evaluation of available **open-source emergency ventilators** in accordance with the global standards and requirements as disclosed by various healthcare organizations such as Health Canada, MHRA, FDA and open-source organizations such as OSHWA.

This document highlights the targeted requirements of emergency ventilators to treat patients with COVID19 Respiratory failure. It is a compilation of various open access information databases made available for the guidance for the rapid development of emergency ventilators by various institutions such as: AAMI, ISO, IEC, MECA, etc.

**THE CONTENT DISCUSSED IN THIS DOCUMENT IS FOR THE DESIGN OF A VENTILATOR TO BE USED EXCLUSIVELY BY TRAINED MEDICAL PROFESSIONALS. ATTEMPTING TO USE A VENTILATOR OR ANY SIMILAR DEVICE WITHOUT MEDICAL SUPERVISION CAN RESULT IN DEATH OR PERMANENT DISABILITY.**

**Use Case:** For the rapid development of emergency use ventilators, under emergency, have disclosed basic requirements for necessary basic operation devices. This document is a compilation of several open access databases and provides a simple guide through the open access availability of such devices across the globe. This document also provides an overview of general open access repository requirements of such devices over several platforms.

**Considerations:** It is understood that the stringent policies for regular ventilators cannot be met in a short span of time, thus, several considerations for the device’s safe development and usability are factored in. The requirements of this document are meant for trained operator use only and thus lay operator requirements are not considered. The documentation and submission protocols across various organizations vary, it is recommended that the user consider them carefully before applying.

**Limitations:** The requirements presented in these documents are solely based on open access updated data from various organizations based on their real time needs and may need to be reviewed based on their requirements. The document addresses the emergency requirements under the Interim Order (IO), Emergency Use Authorization (EUA) and other emergency protocols presented.

1. **Level 1 Evaluation**

It is of critical importance that the data available for open-source projects are readily available in stipulation with the mentioned requirements for the easy reproduction. The developers are suggested to adhere to the following requisites for ensuring the usability of their open-source design.

**1.1 Open-Source Definition**

Open-source projects have several requirements that must be met before they can be declared as open source. The following sections will go through these high-level requirements obtained from the [Open Source Hardware Association (OSHWA)](https://www.oshwa.org/about/) under the  [Creative Commons Attribution-ShareAlike 4.0 International License](http://creativecommons.org/licenses/by-sa/4.0/deed.en_US).

The definition below was obtained from OSHWA and is used for defining what projects are considered open source.

*“Open-source hardware is hardware whose design is made publicly available so that anyone can study, modify, distribute, make, and sell the design or hardware based on that design. The hardware’s source, the design from which it is made, is available in the preferred format for making modifications to it. Ideally, open-source hardware uses readily available components and materials, standard processes, open infrastructure, unrestricted content, and open-source design tools to maximize the ability of individuals to make and use hardware. Open-source hardware gives people the freedom to control their technology while sharing knowledge and encouraging commerce through the open exchange of designs.”*

**1.2 Assessing Open-Source Designs**

OSHWA has provided a [criteria](https://www.oshwa.org/definition/) for open-source hardware designs to comply with the definition of producing an open-source project. They have also produced a [checklist](https://certification.oshwa.org/process.html) that helps certify the project as open source and meet the standard criteria. This document will be using the OSHWA criteria to describe the main features of assessing an open-source project. Furthermore, a viewer of an open-source project will first refer to the documentation of the project to get the required information for building, modifying, or contributing to the project. Hence, the assessment of the open-source project is based primarily on the documentation and information provided.

**1.3 Documentation**

The open-source project must be accompanied by documentation that includes a general description of the components involved for building the project from scratch and its purpose. These include information on design files, bill of materials, instructions and explanations, licensing, and software details. It should also be written in a style for the general audience to understand the content and allow them to reproduce it easily.

**1.3.1 Design Files**

Design files refer to the original source files that are implemented in the design. It is critical that these files use open-source software for their creation to limit the accessibility barrier into the project. But if the files are created using proprietary software, the source files must still be presented to the viewer. Finally, regardless of where these design files are hosted, they need to be organized in a logical manner so that the user understands the significance and purpose of each file. See section 2.4 to for an example of how a project should be structured on GitHub.

The following are some original design file examples obtained from OSHWA:

- 2D drawings or computer-aided design (CAD) files, such as those used to describe two-dimensional laser cut, vinyl cut, or waterjet cut part, in their original format. Example formats: Native 2D design files saved by Corel Draw (.cdr), Inkscape (.svg), Adobe Illustrator (.ai), AutoCAD, etc.
- 3D designs that can be 3D printed, forged, injection molded, extruded, machined, etc. Example formats: Native files saved by SolidWorks (. sldprt. sldasm), Rhino, etc.
- Circuit board CAD files such as capture files (schematics) and printed-circuit board (layout) design files. Example formats: Native files saved by Eagle, Altium, KiCad, gEDA, etc.
- Component libraries (symbol, footprint, fastener, etc.) necessary for native modification of CAD files.
- Additional technical drawings in their original design formats, if required for fabrication of the device.
- Additional artwork that may be used on the device and is included as part of the OSHW release, such as an emblem, or cosmetic overlay in the original design format.

**1.3.2 Bill of Materials**

The bill of materials must include all the parts that make up the hardware unless it is inferred from the design. Include details like part numbers, suppliers, costs, and descriptions for each. Since not every supplier export to every country, it is helpful if alternatives or suitable parts are also mentioned for building the design. It is ideal if the bill of material contains standard components, materials, and procedures to reduce the cost and accessibility barriers. Furthermore, the bill of materials needs to show a connection between the items on the list and how they correspond to the components in the design. All this information should be hosted as a CSV file, Excel file, Google Doc, or other formats that outlines the materials clearly with the required information.

**1.3.3 Instructions and Explanations**

Presenting all the material necessary to build the design is often insufficient for constructing, replicating, or modifying the design. It is important to also include instructions and explanations that guide the user through the process of developing the design.

The following are some pointers from OSHWA on providing the necessary guidance to the viewers.

***Making the hardware***

- To help others make and modify your hardware design, you should provide instructions for going from your design files to the working physical hardware.
- As part of the instructions, it’s helpful to link to datasheets for the components / parts of your hardware and to list the tools required to assemble it.
- If the design requires specialized tools, tell people where to get them.

***Using the hardware***

- Once someone has made the hardware, they need to know how to use it. Provide instructions that explain what it does, how to set it up, and how to interact with it.

***Design rationale***

- If someone wants to modify your design, they’ll want to know why it is the way it is. Explain the plan of the hardware’s design and why you made the specific choices you did.

The instructions could be in a variety of formats, like a wiki, text file, Google Doc, or PDF. But it is helpful to provide original editable files as others might want to modify or improve the instructions as changes are made to the hardware design.

**1.3.4 Licensing**

All open-source projects must present a license that indicates to the viewers that the project can be copied, modified, distributed, or commercialized. The project must also be clear about which elements of the open-source project are open source (i.e., others may want to modify software, documentation, hardware, etc.).

There are many licenses that can be applied to the project, but they need to meet the following criteria obtained directly from OSHWA:

***Derived Works***

- The license shall allow modifications and derived works and shall allow them to be distributed under the same terms as the license of the original work.

- The license shall allow for the manufacture, sale, distribution, and use of products created from the design files, the design files themselves, and derivatives thereof.

***Free retribution***

- The license shall not restrict any party from selling or giving away the project documentation.

- The license shall not require a royalty or other fee for such sale. The license shall not require any royalty or fee related to the sale of derived works.

***Attribution***

- The license may require derived documents, and copyright notices associated with devices, to provide attribution to the licensors when distributing design files, manufactured products, and/or derivatives thereof.

- The license may require that this information be accessible to the end-user using the device normally but shall not specify a specific format of display.

- The license may require derived works to carry a different name or version number from the original design.

***No Discrimination Against Persons or Groups***

- The license must not discriminate against any person or group of persons.

***No Discrimination Against Fields of Endeavor***

- The license must not restrict anyone from making use of the work (including manufactured hardware) in a specific field of endeavor. For example, it must not restrict the hardware from being used in a business, or from being used in nuclear research.

***Distribution of License***

- The rights granted by the license must apply to all to whom the work is redistributed without the need for execution of an additional license by those parties.

***License Must Not Be Specific to a Product***

- The rights granted by the license must not depend on the licensed work being part of a particular product. If a portion is extracted from a work and used or distributed within the terms of the license, all parties to whom that work is redistributed should have the same rights as those that are granted for the original work.

***License Must Not Restrict Other Hardware or Software***

- The license must not place restrictions on other items that are aggregated with the licensed work but not derivative of it. For example, the license must not insist that all other hardware sold with the licensed item be open source, nor that only open-source software be used external to the device.

***License Must Be Technology-Neutral***

- No provision of the license may be predicated on any individual technology, specific part or component, material, or style of interface or use thereof.

**1.3.5 Software Information**

Software required to run the hardware and design must be shared to allow others to modify and run the design. It is recommended that the process required to build the software is well documented including links to third-party libraries, tools, comments, and dependencies. It is also useful to provide an overview of the state of the software (e.g., “stable” or “beta” or “barely-working hack”).

If the licensed design requires software, embedded or otherwise, to operate properly and fulfill its essential functions, then the license may require that one of the following conditions are met according to OSHWA: 

- The interfaces are sufficiently documented such that it could reasonably be considered straightforward to write open-source software that allows the device to operate properly and fulfill its essential functions. For example, this may include the use of detailed signal timing diagrams or pseudocode to clearly illustrate the interface in operation

- The necessary software is released under an OSI-approved open-source license.

**1.4 Hosting Open-Source Projects**

There are a wide variety of digital platforms that can host the open-source project such as GitHub and GitLab. Regardless of the platform that is used for the project, there are some examples of best practices that can be followed.

GitHub has provided the common file maintenance best practices [here](https://guides.github.com/features/wikis/).

1. **Level 2 Evaluation**

This section of the document highlights the compilation of various emergency use ventilator parametric requirements from regulators as mentioned in section 4. It is of prime importance the design used are based on a predicate/precedence model which is already deemed fit for usage. The following requirements are to be fulfilled for satisfying the basic performance of a developed design.

**2.1 Mandatory Parametric Requirements**

**2.1.1 FiO2 & O2**

- Must accommodate the range of 21-100%. *(Health Canada Product Specification)*.
- FiO2 over the range of 21 % (ambient) to 95 % of the source oxygen concentration input to the EUV in no more than 10 % steps. *(EUV)*

**2.1.2 PEEP [*ISO 10651-5, Section 7.1, Health Canada, MHRA, EUV]***

- Must provide a range 5 – 20 cm H2O adjustable in 5 cm H2O increments. *(MHRA Ventilation)*
- PEEP must be maintained during expiration. *(MHRA Ventilation)*
- Inadvertent PEEP: The positive expiratory pressure at the end of the expiratory phase shall not exceed 2 cm H2O.  *(10651-5, 7.1.3)*
- Inadvertent continuing expiratory pressure: Means shall be provided to prevent the build-up of continuing positive pressure from exceeding 2 cm H2O.  *(10651-5, 7.1.4)*
- Set PEEP (i.e., BAP) (5 to 20) cmH2O in no more than 5 cmH2O steps. *(EUV)*

**2.1.3 Flow Rate *[Health Canada]***

- Flow Rates must provide for a gas reservoir to manage peak inspiratory flow rates in the range of 0 – 100 lpm. *(Health Canada Product Specification)*

**2.1.4 Tidal Volume (Vt) [Health Canada, MHRA, EUV]**

- Must accommodate the range of 50-1500 ml (can be scaled back to 1000) as patients VT are based on 4 - 8 ml/kg. *(Health Canada Product Specification).*
- Could provide increments of 50 ml.
- Must have at least one setting of 400ml with +/- 10 ml increments. *(MHRA Ventilation)*
- Upper limit of tidal volume could be set to 800 ml. *(MHRA Ventilation)*
- Tidal volume (350 to 450) ml ±10 % in no more than steps of 50 ml, preferably a lower range of 250 ml and an upper range of 600 ml or 800 ml. *(EUV)*

**2.1.5 Respiratory rate *[ISO 80601 2-80, Health Canada]***

- Resp Rate: 4-45 bpm. (*Health Canada Product Specification)*
- 6RMVS must provide a range 10 - 30 breaths per minute in increments of 2 (only in mandatory mode) that can be set by the user. *(MHRA)*

**2.1.6 Inspiratory: Expiratory Ratio (I: E) *[ISO 10651-3, Section 10, Health Canada, MHRA, EUV]***

- Must provide an adjustable range of 1:1 – 1:4. *(Health Canada Product Specification)*
- Must provide 1:2.0 (i.e. expiration lasts twice as long as inspiration) as the default setting. *(MHRA)*
- The inspiratory and expiratory resistances measured at the patient connection port shall, during spontaneous breathing and normal operation, not exceed 6 cmH20 at flowrates of 60 I/min for adult use, 30 I/min for pediatric use and 5 I/min for neonatal use. *(10651-3, 56.14)*
- I:E ratio (ratio of inspiratory to expiratory time) of 1:2 preferably adjustable from 1:1 to 1:3. *(EUV)*

**2.1.7 Inspiratory Airway Pressure (IAP) *[MHRA, EUV]***

- Where applicable, inspiratory pressure limit (15 to 40) cmH2O preferably adjustable in steps of no more than 5 cmH2O. *(EUV)*
- To help prevent contaminating the environment (and particularly the clinicians), filters need to be placed in the expiratory pathways. Particular attention needs to be placed on the exhaust port. *(EUV)*

**2.1.7.1 Plateau Pressure**

- Plateau pressures should be limited to a maximum of35 cm H2O.

**2.1.7.2 Peak Pressure**

- Peak pressure should be no more than 2 cm H2O greater than plateau pressure. *(MHRA Ventilation).*
- If VCV is used, the user must be able to set inspiratory airway pressure limit in the range at least 15 - 40 cmH2O in at least increments of 5 cmH2O. *(MHRA Ventilation)*
- There must be a mechanical failsafe valve that opens at 80 cmH2O. *(MHRA Ventilation)*

**2.2 Recommended Parametric Requirements**

**2.2.1 Inspiratory resistance during the resuscitator expiratory phase *[CAN/CSA - Z10651 – 5, 7.1.2.2]***

- During the expiratory phase, the pressure at the patient connection port shall not exceed 6 cm H2O below atmospheric pressure at an inspiratory airflow of 60 l/min for resuscitators intended for patients with a body mass greater than 10 kg and of 6 l/min for resuscitators intended for  patients with a body mass up to 10 kg.

**2.2.2 Spontaneous breathing with the gas input pressure outside the rated range *[CAN/CSA - Z10651 – 5, 7.1.2.3 ]***

- When operating with the gas input pressure outside the rated range and during the inspiratory phase, either the resuscitator shall generate a delivered volume and inspiratory time within ± 25 % of that achieved during normal use, or the resuscitator shall be designed to allow spontaneous breathing.
- Under these spontaneous breathing conditions, the pressures below and above atmospheric pressure at the patient connection port shall not exceed 6 cm H2O, at airflows of 30 l/min for resuscitators intended for patients with a body mass greater than 10 kg and of 3 l/min for resuscitators intended for patients with a body mass up to 10 kg.

**2.2.3 Expiratory resistance [CAN/CSA - Z10651 – 5, 7.1.2.4]**

- In the absence of a removable positive end-expiratory pressure (PEEP) valve or with an integral positive end expiratory pressure function set to its minimum value, the pressure at the patient connection port during the expiratory phase shall not exceed 6 cm H2O above atmospheric pressure at an expiratory airflow of 60 l/min for  resuscitators intended for  patients with a body mass greater than 10 kg and of 6 l/min for resuscitators intended for patients with a body mass up to 10 kg.

**2.2.4 Resuscitator dead space and dead space of airway accessories *[CAN/CSA - Z10651 – 5, 7.1.5]***

- The resuscitator dead space shall not exceed 5,5 % of the minimum delivered volume from the resuscitator.

**2.2.5 Inspiratory flow *[CAN/CSA - Z10651 – 5, 7.2.7]***

- A resuscitator with a pre-set flow, intended for use with patients with greater than 40 kg body mass (adult use), when set to deliver > 85 % O2, shall deliver inspiratory flows between 25 l/min and 40 l/min, both on free flow to atmosphere and against a back-pressure of 20 cmH2O. Such resuscitators with operator-adjustable flows shall have a range of adjustment that overlaps this range.

**2.2.6 Threshold pressure for initiation of flow *[CAN/CSA - Z10651 – 5, 7.2.9.2]***

- The pressure at the patient connection port needed to initiate gas flow from the demand valve shall not be numerically greater than 2 cm H2O below atmospheric pressure.

**2.2.7 Peak inspiratory flow *[CAN/CSA - Z10651 – 5, 7.2.9.3]***

- The minimum peak inspiratory flow shall be 100 l/min for at least 2 s, with a pressure at the patient connection port not numerically greater than 8 cm H2O below atmospheric pressure. This flow shall be attained within 250ms.

**2.2.8 Pressure limitation under normal use *[CAN/CSA - Z10651 – 5, 7.2.4]***

The pressure at the patient connection port shall not exceed 60 cm H2O during normal use. A setting for the pressure-limiting device higher than 60hPa may be made available for certain patients, although the selection of such a setting requires medical advice.

1. **Level 3 Evaluation**

**3.1 Regulatory Requirements**

This section addresses the various requirements presented by Health Canada, FDA, MHRA and TGA for the development of emergency ventilators under the emergency declaration.

- - 1. **Health Canada (North America)**

Health Canada announced the [Interim Order (IO)](https://www.canada.ca/en/health-canada/services/drugs-health-products/covid19-industry/interim-order-respecting-clinical-trials-medical-devices-drugs.html) for promoting the rapid development of emergency use medical devices and accessories during COVID-19.

The specifications for the [scaled down ventilators](https://buyandsell.gc.ca/sites/buyandsell.gc.ca/files/non-icu-ventilator-requirements_0.pdf) and [ICU ventilators](https://buyandsell.gc.ca/sites/buyandsell.gc.ca/files/ventilatorspecs2020covid19outbreak.pdf) were also presented for a general overview of the developers. A set of [guidelines](https://www.canada.ca/en/health-canada/services/drugs-health-products/drug-products/announcements/interim-order-importation-sale-medical-devices-covid-19/guidance-medical-device-applications.html) for applicants are issued for easy processing and criteria fulfillment. A [notice](https://www.canada.ca/en/health-canada/services/drugs-health-products/medical-devices/activities/announcements/covid19-notice-importation-sale-ventilators.html#a1) for manufacturers is also to be considered along with the said guidelines.

The list of authorized devices under the Interim Order can be found [here](https://www.canada.ca/en/health-canada/services/drugs-health-products/covid19-industry/medical-devices/authorized/other.html).

- - 1. **FDA (USA)**

The FDA issued an [Emergency Use Authorization (EUA)](https://www.fda.gov/medical-devices/coronavirus-disease-2019-covid-19-emergency-use-authorizations-medical-devices/ventilators-and-ventilator-accessories-euas) which provides a set of guidelines towards the modification and development of emergency use ventilators. It also issued a [letter of authorization](https://www.fda.gov/media/136423/download) for manufacturers to conform with the said practices to ensure device’s usability.

In addition to the requirements of [Fact Sheet for Health Providers](https://www.fda.gov/media/136424/download), [Fact sheet for Patients](https://www.fda.gov/media/136425/download), [Appendix A](https://www.fda.gov/media/136437/download) and [Appendix B](https://www.fda.gov/medical-devices/coronavirus-disease-2019-covid-19-emergency-use-authorizations-medical-devices/ventilators-and-ventilator-accessories-euas#appendixb), the FDA also provides a [template](https://www.fda.gov/media/137172/download) for the application of the EUA.

- - 1. **MHRA (UK)**

Under the emergency, certain [exemptions](https://www.gov.uk/guidance/exemptions-from-devices-regulations-during-the-coronavirus-covid-19-outbreak) have been made by the United Kingdom for the encouragement of emergency ventilator development.

A comprehensive list of [specifications](https://assets.publishing.service.gov.uk/government/uploads/system/uploads/attachment_data/file/879382/RMVS001_v4.pdf) has been released for reference. A list of documents are provided for the [guidance](https://www.gov.uk/government/collections/regulatory-guidance-for-medical-devices) towards various regulatory requirements for manufacturers.

A list of sanctioned emergency use devices is provided [here](https://www.gov.uk/government/publications/medical-devices-given-exceptional-use-authorisations-during-the-covid-19-pandemic/list-of-medical-devices-given-exceptional-use-authorisations).

- - 1. **TGA (Australia)**

The TGA has also addressed the shortage of these devices and has issued a [public notice](https://www.tga.gov.au/behind-news/ventilators-and-other-devices-intended-respiratory-support-covid-19) for the emergency relief of health care officials.

The [specifications](https://www.tga.gov.au/sites/default/files/ventilator-covid-19-use-australia.pdf) are closely knit with those mentioned by the MHRA. Several exemptions have been made for the encouragement of [domestic manufacturing](https://www.tga.gov.au/exemption-enable-domestic-manufacture-and-supply-ventilators#manufacturing) of these devices. One can find several checklists and templates for the easy fulfillment and evaluation in accordance to these requirements.

- 1. **Required Standards**

This document uses references which are under constant evaluations and updates based on the consensus requirements. The information presented in this document may or may not be valid in lieu of post-emergency protocols established.

The following referenced documents are indispensable for the application of this document. It is also to be noted that some of these documents are released in lieu of the global pandemic and may be redacted based on the organization’s decision.

- The **ISO** have made available, a set number of relevant standards for the pandemic for free [here](https://www.iso.org/covid19).
- A set number of **BSI** standards are also made available free [here](https://www.bsigroup.com/en-GB/topics/novel-coronavirus-covid-19/ventilators/).
- **ANSI** has also made available a read only format of standards for the pandemic [here](https://www.ansi.org/news_publications/news_story?menuid=7&articleid=27ba33a0-7482-47c5-b3a7-faa8a55518eb).
- **ASTM** has released a few standards for the manufacturing and production of emergency health care equipment [here](https://www.astm.org/COVID-19/).
- **IEEE** Standards to certain medical devices and printing techniques are listed [here](https://standards.ieee.org/covid-19/index.html).
- The **CSA** also gives a complimentary access to emergency ventilator developers [here](https://www.csagroup.org/news/covid-19-response-standards-handbooks/).
- **AAMI** Standards for the development of emergency ventilators can be found [here](https://www.aami.org/news-resources/covid-19-updates/coronavirus-resources-for-the-field).

Note: The standards mentioned below are under copyright and would need to be purchased for use from their respective sources.

- **AAMI/CR501:** **2020:** *Emergency Use Ventilators (EUVs) Design Guidance*
- **AAMI CR502: 2020:** *End user disclosures for emergency use ventilators (EUVs).*
- **MHRA RMVS001**: *Rapidly Manufactured Ventilator System.*
- **Health Canada:** *Interim Order.*
- **FDA:** *March 24^th^, 2020, Emergency Use Authorization.*
- **TGA:***Therapeutic Goods (Medical Devices—Ventilators) (COVID-19 Emergency) Exemption 2020.*
- **IEC 60601-1: 2012: Medical Electrical Equipment – Part 1**: *General Requirements for Basic Safety and Essential Performance.*
- **IEC 60601-1-2: 2014: Medical Electrical Equipment Part 1-2:** *General Requirements for Basic Safety and Essential Performance – Collateral Standard: Electromagnetic Disturbances – Requirements and Tests.*
- **IEC 60601-1-11: 2015: Medical Electrical Equipment Part 1-11:** *General Requirements for Basic Safety and Essential Performance – Collateral Standard: Requirements for Medical Electrical Equipment and Medical Electrical Systems Used in the Home Healthcare Environment.*
- **Any other applicable collateral/particular standards in the IEC 60601-1:** *2012 family*.
- **IEC 62304: 2015:** *Medical Device Software – Software Life Cycle Processes.*
- **AAMI TIR69: 2017:** *Technical Information Report Risk Management of Radio-Frequency Wireless Coexistence for Medical Devices and Systems*.
- **ANSI/IEEE C63.27: 2017:** *American National Standard for Evaluation of Wireless Coexistence.*
- **AAMI TIR69: 2017:** *Technical Information Report Risk Management of Radio-Frequency Wireless Coexistence for Medical Devices and Systems.*
- **ISO 10993:** **Fifth Edition 2018-08:** Biological Evaluation of Medical Devices - Part 1: *Evaluation and Testing Within a Risk Management Process*.
- **ISO 18562-1 First Edition 2017-03:** *Biocompatibility Evaluation of Breathing Gas Pathways in Healthcare Applications - Part 1: Evaluation and Testing Within a Risk Management Process.*
- **ISO 18562-2 First Edition 2017-03:** *Biocompatibility Evaluation of Breathing Gas Pathways in Healthcare Applications - Part 2: Tests for Emissions of Particulate Matter*.
- **ISO 18562-3 First Edition 2017:** *Biocompatibility Evaluation of Breathing Gas Pathways in Healthcare Applications - Part 3: Tests for Emissions of Volatile Organic Compounds*.
- **ISO 18562-4 First Edition 2017-03:** *Biocompatibility Evaluation of Breathing Gas Pathways in Healthcare Applications - Part 4: Tests for Leachables in Condensate.*
- **ISO 10651-5 First Edition 2006-02-01:** *Lung Ventilators for Medical Use - Particular Requirements for Basic Safety and Essential Performance - Part 5: Gas-Powered Emergency Resuscitators.*
- **ISO 17510 First Edition 2015-08-01:** *Medical devices -- Sleep apnoea breathing therapy -- Masks and application accessories.*
- **ISO 80601-2-12 First Edition 2011-04-15:** Medical Electrical Equipment - Part 2-12*: Particular Requirements for the Safety of Lung Ventilators - Critical Care Ventilators [Including: Technical Corrigendum 1 (2011)]*.
- **ISO 80601-2-13 First Edition 2011-08-11:** Medical Electrical Equipment -- Part 2-13*: Particular Requirements for Basic Safety and Essential Performance of an Anaesthetic Workstation [Including: Amendment 1 (2015) and Amendment 2 (2018)]*.
- **ISO 80601-2-69 First Edition 2014-07-15**: Medical Electrical Equipment - Part 2-69: *Particular Requirements for Basic Safety and Essential Performance of Oxygen Concentrator Equipment.*
- **ISO 80601-2-70 First Edition 2015-01-15:** Medical Electrical Equipment - Part 2-70: *Particular Requirements for Basic Safety and Essential Performance of Sleep Apnoea Breathing Therapy Equipment.*
- **ISO 80601-2-74 First Edition 2017-05:** Medical Electrical Equipment - Part 2-74: *Requirements for Basic Safety and Essential Performance of Respiratory Humidifying Equipment.*
- **ISO 80601-2-79 First Edition 2018-07:** Medical electrical equipment - Part 2-79: *Requirements for Basic Safety and Essential Performance of Ventilatory Support Equipment for Ventilatory Impairment*.
- **ISO 80601-2-80 First Edition 2018-07:** Medical Electrical Equipment - Part 2-80: *Requirements for Basic Safety and Essential Performance of Ventilatory Support Equipment for Ventilatory Insufficiency.*
  - 1. **Guide Through ISO/IEC Standards**

**Note:** *Every regulator has its specific standard requirements to be fulfilled, the extent to which they must be satisfied thus vary. It is advisable that the limitation to the application of the following guidelines is determined based on the desired applicable regulatory requirement as mentioned in section 4.1.*

The standards crucial to the design of an emergency ventilators are in addition to the general medical device standards as mentioned in IEC 60601-1 (Referred to as General Standard, GS). Therefore, it is of utmost importance that the general standards are followed as closely as possible. The following section describes the role of each section in the GS for better understanding and navigation.

The requirements of the sections mentioned below are in alignment of those stated by the AAMI – Emergency Use Ventilation (EUV) Guidelines mentioned in section 4.2.2.

**3.2.1.4 General Requirements**

This section of the GS directs one to follow standard risk management practices throughout the product development phase and requires one to make note of all possible implications of the usage of the device is mapped. The section also highlights the general requirements of the device which must be adhered to with respect to various aspects such as: Components used, Power supplied, parts which come in contact with the patient and their classification.

Other Reference Standards:

- ISO 14971: *Application of Risk Management to Medical Devices*
- ISO 13485: *Medical Devices -Quality Management Systems - Requirements for Regulatory Purposes.*

**3.2.1.5 General Requirements for testing ME Equipment**

This section describes the general physical mandatory tests carried out on the device to ensure a robust, sustainable, and safe device.

Applicable sections of the standard (EUV):

- Mandatory Entirely

**3.2.1.6 Classification of ME Equipment and ME System**

This section of the GS directs one to apply general classification typesets to the device and its various parts as per industry norms. These classifications also imply the application of appropriate risk management processes and usability guidelines.

Applicable sections of the standard (EUV):

- Mandatory Entirely

**3.2.1.7 ME Equipment Identification, marking and documents**

This section of the GS states the labeling and usability requirements for the identification and indication of various traits and accessories as presented by the medical device. This section is an indispensable part of compliance as it identifies various hazards and risks which may arise for the handling of the equipment by the user.

This section also highlights the documentation requirements to demonstrate and present various data pertaining to the performance, risks, hazards, limitations, instruction of use and test results of the device showing the conformity of the device to the said standards.

Other Reference Standards:

- ISO 7010:2003

Applicable sections of the standard (EUV):

- Mandatory subclauses: 7.2 – 7.9

**3.2.1.8 Protection against electrical hazards from ME Equipment**

This section of the GS presents design requirements based on the electrical components and functionality of the device in lieu of the potential hazards which could cause harm to the user, device, patient, and other electrical equipment.

Applicable sections of the standard (EUV):

- Entire section mandatory if necessary.

**3.2.1.9 Protection against mechanical hazards of ME Equipment and ME System**

This section of the GS lists a set of additional mechanical tests and design requirements for various medical devices for a robust and safe usage of the product in lieu of the potential hazards which could cause harm to the user and the patient. It considers factors and mechanical principles by which the device functions such as pneumatics hydraulics, etc.

Applicable sections of the standard (EUV):

- Mandatory Section: 9.3

**3.2.1.10 Protection against unwanted and excessive radiation Hazards**

This section of the General Standards lists a set of tests in accordance with the radiations emitted by the device or any of its components. It considers a general set of common radiations which may compromise the safety of the user, patient, and other equipment in the vicinity of the device.

Applicable sections of the standard (EUV):

- Mandatory Entirely

**3.2.1.11 Protection against excessive temperatures and other Hazards**

This section of the GS highlights the potential hazards and design guidelines which may be incurred due to excessive temperature, reagents used by the device, biocompatibility, interrupted power supply, etc.

Applicable sections of the standard (EUV):

- Mandatory Entirely

**3.2.1.12 Accuracy of controls and instruments and protection against hazardous outputs**

This section of the GS provides design guidelines to incorporate standardized controls over the device parameters and safeguarding the device against hazardous outputs. This section gives an overview of the device’s usability features for ease of use.

This section also highlights the alarm requirements to be fulfilled by the device for the indication and correction of incorrect outputs for immediate correction by the user.

Applicable Sections of the Standard (EUV):

- Mandatory Entirely

**3.2.1.13 Hazardous situations and fault conditions for ME Equipment**

This section of the GS outlines all the general possible hazards which could be encountered by a medical device and the general specifications to correct them. It also describes the testing methods of the device and its components under the single fault condition.

Applicable Sections of the Standard (EUV):

- Mandatory Entirely

**3.2.1.14 Programmable Electrical Medical Systems (PEMS)**

This section of the GS outlines the requirements of the device for being integrated into the PEMS of a health care facility.

Applicable Sections of the Standard (EUV):

- Recommended but not Required.

**3.2.1.15 Construction of ME Equipment**

This section of the GS provides the minimum requirements for design performance which must be fulfilled by the medical device for a safe and robust design.

Applicable Sections of the Standard (EUV):

- Mandatory Entirely

**3.2.1.16 Construction of ME System**

It is important to note that any device or accessory used in conjunction with the medical device is considered a part of the medical system and must therefore comply with the said section of the GS.

This section of the GS outlines the potential hazards caused by the parts and accessories in the medical system and directs the developers to resolve them justly.

Applicable Sections of the Standard (EUV):

- Mandatory Entirely

**3.2.1.17 Electromagnetic Compatibility of Me Equipment and Me Systems**

The medical device/system must not cause any disturbances when functioning around other medical devices. This section of the GS provides a general overview of such requirements and tests to ensure safe operation of the medical device in health care environment.

Applicable Sections of the Standard (EUV):

- Recommended but not Required

The standard 80601-2-80 states the additional requirements for the performance of ventilator devices. These additional requirements must be fulfilled along with those mentioned with the GS Requirements as listed in *Table 4.1.1*. For further information please refer to section 4.2.2.

| **Sections** | **Category** | **Required Clauses from 80601-2-80** |
| --- | --- | --- |
| 3.2.1.5 | General Requirements for testing ME Equipment | - 201.5.101 |
| 3.2.1.7 | ME Equipment Identification, marking and documents | - 201.7.2.4.101 - 201.7.2.13.101 - 201.7.2.101 - 201.7.4.2 - 201.7.4.3 - 201.7.9.1 - *201.7.9.2.1.101 - *201.7.9.2.1.102 - *201.7.9.2.9.101 - 201.7.9.2.8.101 - 201.7.9.2.12 - 201.7.9.2.13.101 - 201.7.9.2.14.101 - 201.7.9.3.1.101 - 201.7.9.3.101 - **201.7.9.2.2.101 |
| 3.2.1.11 | Protection against excessive temperatures and other Hazards | - 201.11.1.2.2 - 201.11.6.6 - 201.11.8.101 - 201.11.8.101.2 |
| 3.2.1.12 | Accuracy of controls and instruments and protection against hazardous outputs | - 201.12.1 - 201.12.1.101 - 201.12.1.102 - 201.12.1 - 201.12.2.101 - 201.12.4 - 201.12.101 |
| 3.2.1.13 | Hazardous situations and fault conditions for ME Equipment | - 201.13.2.101 - 201.13.2.102 |
| 3.2.1.16 | Construction of ME System | - 201.16.1.101 |
|  | Other Required Sections | - 201.101 to 201.108 |
| ** The said clauses are required except that addressing the LAY Operators.*  *** The said clauses are required except for e) and g) sections of the standard.* | | |

*Table 4.1.1: Additional Requirements to General Standards*

- - 1. **Guide Through AAMI**

The Association for the Advancement of Medical Instrumentation (AAMI) formed a consensus report in lieu of the pandemic for the development of [Emergency Use Ventilators (EUV).](https://www.aami.org/docs/default-source/standardslibrary/200410_cr501-2020_rev1-2.pdf?sfvrsn=699e62b7_2)

The report guides one through the limitations and extent of the application of the GS and the ventilation standards of “80601-2-80: Medical Electrical Equipment - Part 2-80: *Particular Requirements for Basic Safety and Essential Performance of Ventilatory Support Equipment for Ventilatory Insufficiency.”*

The organization has also provided an [End User Disclosure](https://www.aami.org/docs/default-source/standardslibrary/200417_cr502-2020_rev1-2.pdf?sfvrsn=72e5fc61_2) guidance document and a draft of [Test Report](https://isotc.iso.org/livelink/livelink?func=ll&objId=21187586&objAction=browse&viewType=1) for emergency ventilators.

- - 1. **Guide Through MECA Checklist**

The Medical Equipment Compliance Associates (MECA) have made available a very detailed set of checklists for [evaluation](https://60601-1.com/wp-content/uploads/2019/04/meca-60601-1-ed3.1-evaluation-package-beta-2018-11-24.pdf), [risk management](https://60601-1.com/wp-content/uploads/2019/04/meca-f-028c-iec-60601-1-2012-risk-management-client-completion-form-0.2revision.pdf), [labeling](https://60601-1.com/wp-content/uploads/2019/04/meca-iec-60601-1-ed3.1-label-manual-checklist-rev4.pdf) and [alarm requirements](https://60601-1.com/wp-content/uploads/2019/04/meca-alarm-standards-cross-reference-2015-04-01.pdf) for the conformance towards the GS.

It also provides guidance towards the evaluation of [critical components](https://60601-1.com/wp-content/uploads/2019/05/critical-components-table-rev35.pdf) for the ventilator design.
