# Supplementary material for: Open ventilator evaluation framework: A synthesized database of regulatory requirements and technical standards for emergency use ventilators from Australia, Canada, UK, and US
Source: HardwareX. 2022 Jan 7;11:e00260. doi: 10.1016/j.ohx.2022.e00260 (PMC8752315; doi:10.1016/j.ohx.2022.e00260)
Supplement: Supplementary data 5 [file mmc5.docx]

**Example Evaluation of the RepRapable Automated Open-Source Bag Valve Mask-based Ventilator**

**General Information**

1. **Manufacturer name:** Michigan Technological University
2. **Device proprietary or brand name:** RepRapable Automated Open Source Bag Valve Mask-based Ventilator
3. **Model Number:** NA
4. **Regulatory Information (Approval/Clearance status in US):** NA
5. **Marketing authorizations in any other country:** NA

*[Please indicate whether the device currently has marketing authorization in another regulatory jurisdiction, such as the European CE Mark, Australian Register of Therapeutic Goods (ARTG) Certificate of Inclusion, Health Canada License, or Japan Pharmaceuticals and Medical Device (PMDA) approval (including certification number, if available).]*

**Reference:** Petsiuk, A.; Tanikella, N.G.; Dertinger, S.; Pringle, A.; Oberloier, S.; Pearce, J. RepRapable Automated Open Source Bag Valve Mask-Based Ventilator. *Preprints* 2020, 2020060318 (doi: 10.20944/preprints202006.0318.v1).

**Methodology:**

The following assessment is carried out based on a pure documented review of the information provided by the developers of the said project. The control parameters and parametric performance are not tested and cannot be confirmed, though some aspects are found to be fulfilled based on the test results provided by the developers. The article summarizes all the findings of the proposed model and provides a thorough walkthrough of every aspect of it. The resources of the project could be found at: <https://osf.io/fjdwz/>. It was found that one would need to physically assess the prototype to carry out Level 2 Evaluation successfully.

**Open Source Evaluation**

| **Verified** | **Requirement** | **Open Source: Documentation** |
| --- | --- | --- |
| ✅ | Must | The hardware must be released with documentation including design files and must allow modification and distribution of the design files. |
| ✅ | Must | Where documentation is not furnished with the physical product, there must be a well-publicized means of obtaining this documentation for no more than a reasonable reproduction cost, preferably downloading via the Internet without charge. |
| ✅ | Must | The documentation must include design files in the preferred format for making changes, for example the native file format of a CAD program. |
| ✅ | Must | Deliberately obfuscated design files must not be allowed |
| ✅ | Must | Intermediate forms analogous to compiled computer code — such as printer-ready copper artwork from a CAD program — are not allowed as substitutes |
| ✅ | Could | The license may require that the design files are provided in fully documented, open format(s). |
| ✅ | Must | The documentation for the hardware must clearly specify what portion of the design, if not all, is being released under the license. |
| ✅ | Should | Your open-source hardware project should include a general description of the hardware’s identity and purpose, written as much as possible for a general audience |

| **Verified** | **Requirement** | **Open Source: License** |
| --- | --- | --- |
| ✅ | Must | If the licensed design requires software, embedded or otherwise, to operate properly and fulfill its essential functions, then the license may require that one of the following conditions are met:   a) The interfaces are sufficiently documented such that it could reasonably be considered straightforward to write open source software that allows the device to operate properly and fulfill its essential functions. For example, this may include the use of detailed signal timing diagrams or pseudocode to clearly illustrate the interface in operation.  b) The necessary software is released under an OSI-approved open source license. |
| ✅ | Must | The license shall allow modifications and derived works and shall allow them to be distributed under the same terms as the license of the original work. |
| ✅ | Must | The license shall allow for the manufacture, sale, distribution, and use of products created from the design files, the design files themselves, and derivatives thereof. |
| ✅ | Must | The license shall not restrict any party from selling or giving away the project documentation |
| ✅ | Must | The license shall not require a royalty or other fee for such sale. The license shall not require any royalty or fee related to the sale of derived works. |
| Inadequate Data for Interpretation | Could | The license may require derived documents, and copyright notices associated with devices, to provide attribution to the licensors when distributing design files, manufactured products, and/or derivatives thereof.  The license may require that this information be accessible to the end-user using the device normally but shall not specify a specific format of display. |
| ✅ | Must | The license may require derived works to carry a different name or version number from the original design |
| ✅ | Must | The license must not discriminate against any person or group of persons |
| ✅ | Must | The license must not restrict anyone from making use of the work (including manufactured hardware) in a specific field of endeavor. For example, it must not restrict the hardware from being used in a business, or from being used in nuclear research. |
| ✅ | Must | The rights granted by the license must apply to all to whom the work is redistributed without the need for execution of an additional license by those parties. |
| Inadequate Data for Interpretation | Must | The rights granted by the license must not depend on the licensed work being part of a particular product. If a portion is extracted from a work and used or distributed within the terms of the license, all parties to whom that work is redistributed should have the same rights as those that are granted for the original work. |
| ✅ | Must | The license must not place restrictions on other items that are aggregated with the licensed work but not derivative of it. For example, the license must not insist that all other hardware sold with the licensed item be open source, nor that only open source software be used external to the device. |
| Inadequate Data for Interpretation | Must | No provision of the license may be predicated on any individual technology, specific part or component, material, or style of interface or use thereof. |
| ✅ | Must | Note that the definition of open-source hardware specifies that you must allow modification and commercial re-use of your design, so do not use licenses with a no-derivatives or non-commercial clause. |
| ✅ | Should | When licensing your project, keep in mind that someone who makes a derivative of your hardware will probably also want to build on your software, instructions, and other documentation; you should license not just the hardware design files but also these other elements of your project. |

| **Verified** | **Requirement** | **Open Source: Original and Auxiliary Design Files** |
| --- | --- | --- |
| ✅ | Must | Share the original source files that you would use to make modifications to the hardware’s design. Ideally, your open-source hardware project would be designed using a free and open-source software application, to maximize the ability of others to view and edit it.   Examples of Original Design Files include: • 2D drawings or computer-aided design (CAD) files, such as those used to describe two-dimensional laser cut, vinyl cut, or water-jet cut part, in their original format. Example formats: Native 2D design files saved by Corel Draw (.cdr), Inkscape (.svg), Adobe Illustrator (.ai), AutoCAD, etc. • 3D designs that can be 3D printed, forged, injection molded, extruded, machined, etc. Example formats: Native files saved by SolidWorks (. sldprt, sldasm), Rhino, etc. • Circuit board CAD files such as capture files (schematics) and printed-circuit board (layout) design files. Example formats: Native files saved by Eagle, Altium, KiCad, gEDA, etc. • Component libraries (symbol, footprint, fastener, etc.) necessary for native modification of CAD files. • Additional technical drawings in their original design formats, if required for fabrication of the device. • Additional artwork that may be used on the device and is included as part of the OSHW release, such as an emblem, or cosmetic overlay in the original design format.  Examples of alternative formats that could constitute original design files under special circumstances include:  • Hand-coded G-code for a machined part. (G-code)  • Scans of hand-drawn blueprints. (JPEG)  • Detailed 3D scans of a hand-carved resin-casting mold. (STL)  • Mask pattern for etching a single-side circuit board, as drawn in MS Paint. (PNG) |
| ✅ | Must | Hardware design files created in proprietary programs and stored in proprietary formats are still required to be shared. They are the very files that someone will need in order to contribute changes to a given design. |
| ✅ | Should | Organize files in a logical way; comment complex aspects; note any unusual manufacturing procedures; etc. |
| ✅ | Should | Original design files need to be presented in their native file format as well as in more accessible formats so that it can be opened or imported by other CAD programs.  Examples of auxiliary design files include: • 2D drawings or CAD files, in a 2D export or interchange format. Example formats: DXF, SVG • 2D drawings or CAD files, in an easily viewable 2D export format. Example formats: PDF, JPEG, PNG, etc. (Where possible, vector formats are preferred over bitmap formats.) • 3D designs or CAD files, in a 3D export or interchange format. Example formats: STEP, IGES • 2D or 3D designs in manufacturing-ready export formats Example formats: G-code, STEP-NC, STL, AMF • Circuit board design files in export or interchange formats. Example formats: EDIF, Open JSON • Circuit board designs in manufacturing-ready formats Example formats: Gerber RS-274X, Excellon • Additional technical drawings in their original formats, if required for fabrication of the device, in a commonly-readable format such as PDF. • Additional artwork, for example different colored skins for an instrument panel. |
| ✅ | Should | It is also helpful to provide ready-to-view outputs that can easily be viewed by end users who wish to understand (but not necessarily modify) the design. For example, a PDF of a circuit board schematic, or an STL of a 3D design. These auxiliary design files allow people to study the design of the hardware, and sometimes even fabricate it, even without access to particular proprietary software packages. However, note that auxiliary design files are never allowed as substitutes for original design files |

| **Verified** | **Requirement** | **Open Source: Bill of Materials** |
| --- | --- | --- |
| ✅ | Should | While it might be possible to infer from the design files which parts make up a piece of hardware, it is important to provide a separate bill of materials. This can be a spreadsheet (e.g. CSV, XLS, Google Doc) or simply a text file with one part per line. If your CAD package has integrated or add-on BOM management tools, those are also a good option. (Examples include the built-in tools in SolidWorks and bom-ex for Eagle.) |
| ✅ | Should | Useful things to include in the bill of materials are part numbers, suppliers, costs, and a short description of each part. |
| ✅ | Should | Make it easy to tell which item in the bill of materials corresponds to which component in your design files: use matching reference designators in both places, provide a diagram indicating which part goes where, or otherwise explain the correspondence. |

| **Verified** | **Requirement** | **Open Source: Software and Firmware** |
| --- | --- | --- |
| ✅ | Should | You should share any code or firmware required to operate your hardware. This will allow others to use it with their hardware or modify it along with their modifications to your hardware. Document the process required to build your software, including links to any dependencies (e.g. third-party libraries or tools). In addition, it’s helpful to provide an overview of the state of the software (e.g. “stable” or “beta” or “barely-working hack”). |

| **Verified** | **Requirement** | **Open Source: Photos** |
| --- | --- | --- |
| ✅ | Should | Photos help people understand what your project is and how to put it together. It’s good to publish photographs from multiple viewpoints and at various stages of assembly. If you don’t have photos, posting 3D renderings of your design is a good alternative. Either way, it’s good to provide captions or text that explain what’s shown in each image and why’s it’s useful. |

| **Verified** | **Requirement** | **Open Source: Instructions and Other Explanations** |
| --- | --- | --- |
| ✅ | Should | Making the hardware: To help others make and modify your hardware design, you should provide instructions for going from your design files to the working physical hardware. As part of the instructions, it’s helpful to link to datasheets for the components / parts of your hardware and to list the tools required to assemble it. If the design requires specialized tools, tell people where to get them. |
| ✅ | Should | Using the hardware: Once someone has made the hardware, they need to know how to use it. Provide instructions that explain what it does, how to set it up, and how to interact with it. |
| ✅ | Should | Design rationale: If someone wants to modify your design, they’ll want to know why it is the way it is. Explain the overall plan of the hardware’s design and why you made the specific choices you did. |
| ✅ | Should | Keep in mind that these instructions may be read by someone whose expertise or training is different from yours. As much as possible, try to write to a general audience, and check your instructions for industry jargon, be explicit about what you assume the user knows, etc. |
| ✅ | Should | The instructions could be in a variety of formats, like a wiki, text file, Google Doc, or PDF. Remember, though, that others might want to modify your instructions as they modify your hardware design, so it’s good to provide the original editable files for your documentation, not just output formats like PDF. |

| **Verified** | **Requirement** | **Open Source: Designing your Hardware** |
| --- | --- | --- |
| ✅ | Should | Use free and open-source software design (CAD) tools where possible. If that’s not feasible, try to use low-cost and/or widely used software packages. |
| ✅ | Should | Use standard and widely available components, materials, and production processes. Try to avoid parts that aren’t available to individual customers or processes that require expensive setup costs. |

| **Verified** | **Requirement** | **Open Source: Hosting and Distributing** |
| --- | --- | --- |
| Inadequate Data for Interpretation | Should | All files (design, bill-of-materials, assembly instructions, code, etc.) should be version controlled where possible. |
| ✅ | Should | Provide links to the source (original design files) for your hardware on the product itself, its packaging, or its documentation. |
| ✅ | Should | Make it easy to find the source (original design files) from the website for a product. |
| Inadequate Data for Interpretation | Should | Label the hardware with a version number or release date so that people can match the physical object with the corresponding version of its design files. |
| Inadequate Data for Interpretation | Should | Use the open-source hardware logo on your hardware. Do so in a way that makes it clear which parts of the hardware the logo applies to (i.e., which parts are open source). |
| ✅ | Should | In general, clearly indicate which parts of a product are open source (and which aren’t). |
| ✅ | Should | Don’t refer to hardware as open source until the design files are available. If you plan on open sourcing the product in the future, say that instead. |

| **Verified** | **Requirement** | **Open Source: GitHub** |
| --- | --- | --- |
| ✅ | Should | Include your project name in the README. Your project’s name is the first thing people will see upon scrolling down to your README and is included upon creation of your README file. |
| ✅ | Should | Include a description in the README. A good description is clear, short, and to the point. Describe the importance of your project, and what it does. |
| **❌** | Should | Optionally, include a table of contents in order to allow other people to quickly navigate especially long or detailed READMEs. |
| ✅ | Should | Include an installation section in the README. Tell other users how to install your project locally. Optionally, include a gif to make the process even more clear for other people. |
| ✅ | Should | Include a usage section in the README, in which you instruct other people on how to use your project after they’ve installed it. This would also be a good place to include screenshots of your project in action. |
| **❌** | Should | Larger projects often have sections on contributing to their project in the README, in which contribution instructions are outlined. Sometimes, this is a separate file. If you have specific contribution preferences, explain them so that other developers know how to best contribute to your work. |
| ✅ | Should | Include a section for credits in the README in order to highlight and link to the authors of your project. |
| ✅ | Should | Include a section for the license of your project in the README file |
| ✅ | Should | Your README should contain only the necessary information for developers to get started using and contributing to your project. Longer documentation is best suited for wikis. |
| ✅ | Should | Finally, open source projects use the following tools to organize discussion. Reading through the archives will give you a good picture of how the community thinks and works. Ask, does the project has one or all the following:   Issue tracker: Where people discuss issues related to the project.   Pull requests: Where people discuss and review changes that are in progress.  Discussion forums or mailing lists: Some projects may use these channels for conversational topics. Others use the issue tracker for all conversations.  Synchronous chat channel: Some projects use chat channels (such as Slack or IRC) for casual conversation, collaboration, and quick exchanges. |

| **Verified** | **Requirement** | **Open Source: Building on Open-Source Hardware** |
| --- | --- | --- |
| NA | Must | Respect the trademarks of others |
| NA | Should | Share your changes and improvements with the creator of the original hardware. |
| NA | Should | Be emotionally prepared to allow your project to be copied (unless your trademark is violated, then act according to trademark law). |
| NA | Should | While direct commercial use of existing open source hardware designs is explicitly allowed, it is better — when possible — to make useful improvements to the design and to release that improved version as open source hardware. |
| NA | Should | Consider registering the project under your trademark. Trademark law prevents other people from using your brand name or logo on their own hardware. But it does not stop them from copying your product’s function so long as they do not copy its trademark-protected design. |

***Open Source Hardware Certification Checklist***

Link: <https://certification.oshwa.org/process.html>

| **Verified** | **HARDWARE** |
| --- | --- |
| ✅ | Have you provided links to your original design files for your hardware on the product itself or its documentation? |
| ✅ | Have you made it easy to find your original design files from the website for a product? |
| ✅ | Have you clearly indicated which parts of a product are open source (and which aren’t)? |
| ✅ | Have you applied an open source license to your hardware? |
|  | After certification, remember to:   - Label your hardware with a version number or release date, so people can match the physical object with the corresponding version of its design files. - Use the OSHWA certification mark logo on your hardware. Do so in a way that makes it clear which parts of the hardware the logo applies to (i.e. which parts are open-source). |

| **Verified** | **SOFTWARE** |
| --- | --- |
| ✅ | Have you made your project’s software publicly available? |
| ✅ | Have you applied an open source license to your software? |
| **❌** | The OSHWA definition requires all software that is necessary for the operation of your hardware to be licensed under an [OSI-approved license](https://opensource.org/licenses). |

| **Verified** | **DOCUMENTATION** |
| --- | --- |
| ✅ | Have you made your original design files publicly available? |
| ✅ | Have you made your any auxiliary design files publicly available? (optional) |
| ✅ | Have you made a bill of materials publicly available? |
| ✅ | Have you made photos of your product at various stages of assembly publicly available? (optional) |
| ✅ | Have you made any instructions or other explanations publicly available? (optional) |
| ✅ | Have you properly licensed your design files so that others may reproduce or build upon them? |

| **Verified** | **BRANDING** |
| --- | --- |
| NA | Does your product have any branding elements? |
| NA | Have you chosen original brand names, product names, logos, and product designs (if applicable)? |
| NA | Have you read the [USPTO Trademark Basics Guide](https://www.uspto.gov/trademarks-getting-started/trademark-basics)? |
| NA | Have you consulted with a trademark lawyer, or registered your trademarks on your own? |

**Summary:**

The level 1 assessment of the RepRapable Emergency Ventilator was conducted based on the resources provided. The following conclusions could be drawn after the 1^st^ Assessment:

- The Project is well structured and is readable for developers. It could satisfy most of the open-source repository requirements for a good open-source product.

**Limitations:**

The Level 1 assessment though provides a thorough guideline towards the repository elements which are to be included in an open-source project, the functionality of these elements still must be determined by practical application of the provided Information.

It is also found that the information provided by developers may be in any form and hence tracking it down towards the completion of the assessment could be difficult.

**Limitation of Level 2 Assessment:**

- The project doesn’t explicitly mention the parametric conformance of their device towards a particular standard.
- A wide range of elements for the assessment can only be carried out by operating and testing the device in person.

Assessing a design on the basis of information provided by the developer cannot be achieved. The framework only identifies the models which are worthy to further investigate or invest towards their development. It highlights the gaps which must be addressed by the developers for an easy assessment.

**Parametric Compliance**

The following data should be tested and verified based on the provided test reports and the replication of the model of the proposed design in person by the clinician.

| **Verified** | **Section** | **Comment** | **Parameter** |  |
| --- | --- | --- | --- | --- |
| **FiO2** | | | |  |
| Inadequate Data for Interpretation |  |  | Must accommodate the range of 21-100%. |  |
| Inadequate Data for Interpretation |  |  | FiO2 over the range of 21 % (ambient) to 95 % of the source oxygen concentration input to the EUV in no more than 10 % steps. |  |
| **PEEP** | | | |  |
| ✅ |  |  | Must provide a range 5 – 20 cm H2O adjustable in 5 cm H2O increments. |  |
| Inadequate Data for Interpretation |  |  | PEEP must be maintained during expiration. |  |
| Inadequate Data for Interpretation |  |  | Inadvertent PEEP: The positive expiratory pressure at the end of the expiratory phase shall not exceed 2 cm H2O. |  |
| Inadequate Data for Interpretation |  |  | Inadvertent continuing expiratory pressure: Means shall be provided to prevent the build-up of continuing positive pressure from exceeding 2 cm H2O. |  |
| Inadequate Data for Interpretation |  |  | Set PEEP (i.e. BAP) (5 to 20) cmH2O in no more than 5 cmH2O steps. |  |
| **Flowrate** | | | |  |
| Inadequate Data for Interpretation |  |  | Flow Rates must provide for a gas reservoir to manage peak inspiratory flow rates in the range of 0 - 100lpm. |  |
| **Tidal Volume** | | | | |
| ✅ |  |  | Must accommodate the range of 50-1000 ml (can be scaled back to 800) as patients VT are based on 4 - 8 ml/kg. |  |
| Inadequate Data for Interpretation |  |  | Could provide increments of 50 ml. |  |
| Inadequate Data for Interpretation |  |  | Must have at least one setting of 400ml with +/- 10 ml increments. |  |
| ✅ |  |  | Upper limit of tidal volume could be set to 800 ml. |  |
| Inadequate Data for Interpretation |  |  | Tidal volume (350 to 450) ml ±10 % in no more than steps of 50 ml, preferably a lower range of 250 ml and an upper range of 600 ml or 800 ml. | |
| **Minute Ventilation** | | | | |
| Inadequate Data for Interpretation |  |  | Must be able to accommodate the range of 5 – 10 L/minute. | |
| **Respiratory Rate** | | | | |
| ✅ |  |  | Resp Rate: 4-45 bpm | |
| Inadequate Data for Interpretation |  |  | Must provide a range 10 - 30 breaths per minute in increments of 2 (only in mandatory mode) that can be set by the user. | |
| **Inspiratory/Expiratory Ratio** | | | | |
| ✅ |  |  | Must provide an adjustable range of 1:1 – 1:4. | |
| Inadequate Data for Interpretation |  |  | Must provide 1:2.0 (i.e. expiration lasts twice as long as inspiration) as the default setting. | |
| Inadequate Data for Interpretation |  |  | The inspiratory and expiratory resistances measured at the patient connection port shall, during spontaneous breathing and normal operation, not exceed 6 cmH20 at flowrates of 60 I/min for adult use, 30 I/min for pediatric use and 5 I/min for neonatal use. | |
| ✅ |  |  | I:E ratio (ratio of inspiratory to expiratory time) of 1:2 preferably adjustable from 1:1 to 1:3. | |
| **Inspiratory Airway Pressure** | | | | |
| Inadequate Data for Interpretation |  |  | Where applicable, inspiratory pressure limit (15 to 40) cmH2O preferably adjustable in steps of no more than 5 cmH2O. | |
| Inadequate Data for Interpretation |  |  | To help prevent contaminating the environment (and particularly the clinicians), filters need to be placed in the expiratory pathways. Particular attention needs to be placed on the exhaust port. | |
| **❌** |  |  | Plateau pressures should be limited to a maximum of35 cm H2O. | |
| **❌** |  |  | Peak pressure should be no more than 2 cm H2O greater than plateau pressure. | |
| Inadequate Data for Interpretation |  |  | If VCV is used, the user must be able to set inspiratory airway pressure limit in the range at least 15 - 40 cmH2O in at least increments of 5 cmH2O. | |
| Inadequate Data for Interpretation |  |  | There must be a mechanical failsafe valve that opens at 80 cmH2O. | |
| **Inspiratory resistance during the resuscitator expiratory phase** | | | | |
|  |  |  | During the expiratory phase, the pressure at the patient connection port shall not exceed 6 cm H2O below atmospheric pressure at an inspiratory airflow of 60 l/min for resuscitators intended for patients with a body mass greater than 10 kg and of 6 l/min for  resuscitators intended for  patients with a body mass up to 10 kg. | |
| **Spontaneous breathing with the gas input pressure outside the rated range** | | | | |
|  |  |  | When operating with the gas input pressure outside the rated range and during the inspiratory phase, either the resuscitator shall generate a delivered volume and inspiratory time within ± 25 % of that achieved during normal use, or the resuscitator shall be designed to allow spontaneous breathing.  Under these spontaneous breathing conditions, the pressures below and above atmospheric pressure at the patient connection port shall not exceed 6 cm H2O, at airflows of 30 l/min for resuscitators intended for patients with a body mass greater than 10 kg and of 3 l/min for  resuscitators intended for patients with a body mass up to 10 kg. | |
| **Expiratory resistance** | | | | |
|  |  |  | In the absence of a removable positive end-expiratory pressure (PEEP) valve or with an integral positive end expiratory pressure function set to its minimum value, the pressure at the patient connection port during the expiratory phase shall not exceed 6 cm H2O above atmospheric pressure at an expiratory airflow of 60 l/min for  resuscitators intended for  patients with a body mass greater than 10 kg and of 6 l/min for resuscitators intended for patients with a body mass up to 10 kg. | |
| **Resuscitator dead space and dead space of airway accessories** | | | | |
|  |  |  | The resuscitator dead space shall not exceed 5.5 % of the minimum delivered volume from the Resuscitator. | |
| **Inspiratory flow** | | | | |
|  |  |  | A resuscitator with a pre-set flow, intended for use with patients with greater than 40 kg body mass (adult use), when set to deliver > 85 % O2, shall deliver inspiratory flows between 25 l/min and 40 l/min, both on free flow to atmosphere and against a back-pressure of 20 cmH2O.  Such resuscitators with operator-adjustable flows shall have a range of adjustment that overlaps this range. | |
| **Threshold pressure for initiation of flow** | | | | |
|  |  |  | The pressure at the patient connection port needed to initiate gas flow from the demand valve shall not be numerically greater than 2 cm H2O below atmospheric pressure. | |
| **Peak inspiratory flow** | | | | |
|  |  |  | The minimum peak inspiratory flow shall be 100 l/min for at least 2 s, with a pressure at the patient connection port not numerically greater than 8 cm H2O below atmospheric pressure. This flow shall be attained within 250ms. | |
| **Pressure limitation under normal use** | | | | |
|  |  |  | The pressure at the patient connection port shall not exceed 60 cm H2O during normal use. A setting for the pressure-limiting device higher than 60hPa may be made available for certain patients, although the selection of such a setting requires medical advice. | |

| **Verified** | **Section** | **Comment** | **Alarm Description** |
| --- | --- | --- | --- |
| **Monitoring and Alarm Conditions** | | | |
| Partially Fulfilled |  |  | Must have alarms to indicate the following:   - Gas failures - Electrical supply failures - Machine has switched off during ventilation - Exceeding inspiratory airway pressure - Inspiratory and PEEP pressure not achieved (disconnection alarm) - Tidal volume not achieved or exceeded - If pressure support mode is provided, there must be real time confirmation of each patient breath and an alarm if below acceptable range - Where PCV is used, the user must be able to set patient specific upper and lower tidal volume alerts to alert to the need to adjust pressure. |
| **Low Airway Pressure Alarm Condition** | | | |
| Inadequate Data for Interpretation |  |  | Must have an alarm system to indicate with a medium priority when low airway pressure alarm limit has been met. |
| **High Pressure Alarm Condition and Protection Device** | | | |
| Inadequate Data for Interpretation |  |  | Must have an alarm system to indicate with a high priority when high airway pressure alarm limit has been met under the conditions outlined in this standard. |
| **Hypoventilation Alarm Condition** | | | |
| Inadequate Data for Interpretation |  |  | Must have an alarm system to indicate hypoventilation |
| **Alarm Conditions** | | | |
| Inadequate Data for Interpretation |  |  | Must have the alarm system indicate when power is being switched over to the internal power supply with the appropriate timings specified in this clause. |
| **Independence of ventilation control function and related Risk Control measures** | | | |
| **❌** |  |  | Must have risk control measures to prevent the failure of the alarm system |

**Declaration of Conformance**

| **Verified** | **Standard** |
| --- | --- |
|  | **IEC 60601-1: 2012**: *Medical Electrical Equipment – Part 1: General Requirements for Basic Safety and Essential Performance* |
|  | **IEC 60601-1-2: 2014**: *Medical Electrical Equipment Part 1-2: General Requirements for Basic Safety and Essential Performance – Collateral Standard: Electromagnetic Disturbances – Requirements and Tests* |
|  | **IEC 60601-1-11: 2015**: *Medical Electrical Equipment Part 1-11: General Requirements for Basic Safety and Essential Performance – Collateral Standard: Requirements for Medical Electrical Equipment and Medical Electrical Systems Used in the Home Healthcare Environment* |
|  | Any other applicable collateral/particular standards in the IEC 60601-1: 2012 family |
|  | **IEC 62304: 2015**: *Medical Device Software – Software Life Cycle Processes* |
|  | **AAMI TIR69: 2017:** *Technical Information Report Risk Management of Radio- Frequency Wireless Coexistence for Medical Devices and Systems* |
|  | **ANSI/IEEE C63.27: 2017:** *American National Standard for Evaluation of Wireless Coexistence* |
|  | **AAMI TIR69: 2017:** *Technical Information Report Risk Management of Radio- Frequency Wireless Coexistence for Medical Devices and Systems* |
|  | **ISO 10993: Fifth Edition 2018-08:** Biological Evaluation of Medical Devices - Part 1: Evaluation and Testing Within a Risk Management Process |
|  | **ISO 18562-1 First Edition 2017-03:** *Biocompatibility Evaluation of Breathing Gas Pathways in Healthcare Applications - Part 1: Evaluation and Testing Within a Risk Management Process* |
|  | **ISO 18562-2 First Edition 2017-03:** *Biocompatibility Evaluation of Breathing Gas Pathways in Healthcare Applications - Part 2: Tests for Emissions of Particulate Matter* |
|  | **ISO 18562-3 First Edition 2017:** *Biocompatibility Evaluation of Breathing Gas Pathways in Healthcare Applications - Part 3: Tests for Emissions of Volatile Organic Compounds* |
|  | **ISO 18562-4 First Edition 2017-03:** *Biocompatibility Evaluation of Breathing Gas Pathways in Healthcare Applications - Part 4: Tests for Leachables in Condensate* |
|  | **ISO 10651-5 First Edition 2006-02-01:** *Lung Ventilators for Medical Use - Particular Requirements for Basic Safety and Essential Performance - Part 5: Gas-Powered Emergency Resuscitators* |
| ✅ | **ISO 80601-2-12 First Edition 2011-04-15:** *Medical Electrical Equipment - Part 2-12: Particular Requirements for the Safety of Lung Ventilators - Critical Care Ventilators [Including: Technical Corrigendum 1 (2011)]* |
|  | **ISO 17510 First Edition 2015-08-01:** *Medical devices -- Sleep apnoea breathing therapy -- Masks and application accessories* |
|  | **ISO 80601-2-13 First Edition 2011-08-11:** *Medical Electrical Equipment -- Part 2-13: Particular Requirements for Basic Safety and Essential Performance of an Anaesthetic Workstation [Including: Amendment 1 (2015) and Amendment 2 (2018)]* |
|  | **ISO 80601-2-69 First Edition 2014-07-15:** *Medical Electrical Equipment - Part 2-69: Particular Requirements for Basic Safety and Essential Performance of Oxygen Concentrator Equipment* |
|  | **ISO 80601-2-70 First Edition 2015-01-15:** *Medical Electrical Equipment - Part 2-70: Particular Requirements for Basic Safety and Essential Performance of Sleep Apnoea Breathing Therapy Equipment* |
|  | **ISO 80601-2-74 First Edition 2017-05:** *Medical Electrical Equipment - Part 2-74: Particular Requirements for Basic Safety and Essential Performance of Respiratory Humidifying Equipment* |
|  | **ISO 80601-2-79 First Edition 2018-07:** *Medical electrical equipment - Part 2-79: Particular Requirements for Basic Safety and Essential Performance of Ventilatory Support Equipment for Ventilatory Impairment* |
|  | **ISO 80601-2-80 First Edition 2018-07:** *Medical Electrical Equipment - Part 2-80: Particular Requirements for Basic Safety and Essential Performance of Ventilatory Support Equipment for Ventilatory Insufficiency* |

**Device Specifications and Instructions for Ventilators and Accessories**

For devices for delivering ventilatory support, sponsors should provide specific information and instructions regarding the device’s:

| **Verification** | **Description** |
| --- | --- |
|  | Available ventilation modes |
|  | Patient interfaces |
|  | Ventilatory parameter ranges (e.g., maximum inspiratory pressure, positive end-expiratory pressure, respiration rate, flow, delivered tidal volume, triggering, etc.) |
|  | Battery specifications (if applicable), including runtime |
|  | How users are notified of device battery status (e.g., alarms), and expected use life that is supported by testing. |
|  | For devices with external or replaceable internal batteries, the sponsor should provide information regarding chemistry, including information regarding design, capacity, and software and/or hardware risk mitigations for overcharging alarms, and information regarding conformance to applicable standards (e.g., IEC62311 for rechargeable batteries or IEC 60086-4 for non-rechargeable batteries for lithium-ion technology) |
|  | Description of the device’s alarm functionality, including a listing of all alarm conditions and the associated default settings and limits |
|  | Description of the device’s sensors and monitored parameters (including device parameters or patient parameters, as applicable) |
|  | For ventilator accessories sponsors should provide specific information and instructions (as applicable), regarding the device’s:   - Connection dimensional characteristics (i.e., per ISO 5356-1) types (e.g. single limb with active exhalation, dual limb) - Compensating controls |

**Reprocessing and Shelf-life Information**

Sponsors of ventilators, ventilator tubing connectors, and ventilator accessories should provide the following information and instructions regarding device reprocessing.

| **Verification** | **Description** |
| --- | --- |
|  | Instructions on how to reprocess reusable accessories, including filters and sensors. (refer to FDA’s guidance Reprocessing Medical Devices in Health Care Settings: Validation Methods and Labeling) |
|  | A list of all components—both internal and external to the ventilator—that can contact patient-expired gases or may become contaminated with patient bodily fluids. Such components may include, but are not limited to: the expiratory module, flow sensors, pressure sensors, humidifier, patient circuit, carbon dioxide module sensor.  The list should specify whether the device components are intended for single use or are reusable. This applies to both patient-contacting components, as well as components that may otherwise come in contact or be contaminated with patient-expired gases or bodily fluids |
|  | Information regarding device shelf-life |

**Facility Requirements (as applicable)**

As applicable, sponsors of ventilators, ventilator tubing connectors, and ventilator accessories should provide the following information:

| **Verification** | **Description** |
| --- | --- |
|  | Gas input connection type (e.g., Diameter Index Safety System (DISS), NIST) |
|  | Gas type (e.g., air, oxygen), including information regarding input pressures and flow rates |
|  | Gas source (e.g., internal blower, wall-source) |
|  | Environmental controls to reduce transmission (e.g., negative pressure) |

**Labelling Requirements for conditions of use**

| **Verification** | **Description** |
| --- | --- |
|  | The device’s labeling includes the device’s specifications (including ventilatory parameters), information regarding alarms (e.g., disconnect, EtCO2 alarms, etc.), device reprocessing instructions, and other instructions described above as applicable. |
| **The Fact Sheet for Healthcare Providers administering the device includes the following:** | |
|  | A statement that FDA has authorized the emergency use of the device; |
|  | A description of the significant known and potential benefits and risks of the emergency |
|  | Information regarding the available alternatives to the device, including benefits and risks of the available alternatives |
| **The Fact Sheet for Patients to whom the device is administered includes the following:** | |
|  | A statement that FDA has authorized the emergency use of the device; |
|  | A description of the significant known and potential benefits and risks of the emergency use of the device, and of the extent to which such benefit and risks are unknown; and |
|  | Information regarding the individual’s option to accept or refuse administration of the device; of the consequence, if any, of refusing administration of the device; and of the available alternatives to the device, including the benefits and risks of the available alternatives. |
|  | A single ventilator fitted with the Vent Splitter can be used for multiple patients for ventilatory support during the COVID-19 pandemic when individual ventilators are not available or pre-emptively to increase the potential of single-use ventilators permitting mechanical ventilation for multiple patients simultaneously; |
|  | A description of the recommended use options/configuration (e.g. 2 splitters that can provide 2 ventilatory circuits (2 patients), 4 splitters that can provide 3 ventilatory circuits (3 patients) or 6 splitters that can provide 4 ventilatory circuits (4 patients); recommendations regarding the need for extra long tubing if needed to position patients in a manner that allows access to the patients and the ventilator; recommendations regarding free gas flow (FGF) requirements for oxygen when the ventilator used for multiple patients. |
|  | The pressure control mode is recommended when more than one circuit is added to the ventilator |
| **Labelling Requirements** | |
|  | The single ventilator fitted with the Vent Splitters will provide each patient with the same level of pressure support, the same rate of respiration, the same inspiratory/expiratory ratio, the same FiO2, the same level of PEEP, etc. |
|  | Because the single ventilator provides similar ventilatory support to all patients, it is important to size match patients |
|  | Cautionary statement regarding the need for paralysis and sedation, and the need for additional infusion pumps to administer these agents, to avoid dyssynchronous breathing and system alarming from bucking and coughing; |
|  | Cautionary statement regarding the need for additional infusions pumps |
|  | Because the single ventilator provides similar ventilatory support to all patients, it is also important to select, to the extent possible, patients with similar underlying lung physiology, lung compliance, and ventilatory requirements, so that one system can generally, meet each patient’s needs, as they await individualized ventilators; |
|  | A description of recommended approach to patient monitoring, e.g. each patient should be assessed frequently clinically, at a minimum of 15-30 minute intervals, including vital signs, oxygen saturation level, end tidal Co2, examinations of the chest for bilateral air movement, and, if indicated, assessments of arterial blood gas findings to assure clinical stability on the shared system; close monitoring of all patients will be critical since they will likely be paralyzed and sedated. |
|  | If the shared ventilator alarms for any reason, clinical assessments of each patient are indicated immediately in order to determine which patient is triggering the alarm. The ventilator cannot indicate which patient is triggering the alarm. Providers need to assess all patients, consider suctioning and proper tube placement, and disconnect any unstable patient, considering mechanical bagging if necessary; |
|  | Potential infectious complications from sharing one ventilator have not been studied, and therefore caution is advised. If patients share the same infection, the single ventilator for multiple patients is a viable short-term management option. Each patient’s is individualized with in-line filters designed to filter out viruses and/or bacteria and to protect the ventilator from contamination. |

**Continuous Ventilator Splitters (Adapters for Multiplexing)**

Engineering and manufacturing considerations for a ventilator circuit adapter for multiplexing certain continuous ventilators intended for use in a healthcare facility (21 CFR 868.5895 and product code CBK (ventilator, continuous, facility use)) are set forth below.

Sponsors should provide a description or discussion demonstrating their assessment of these considerations

| **Verification** | **Engineering and Manufacturing Considerations:** |
| --- | --- |
|  | Material properties and high polymeric crosslinking/conversion |
|  | Material strength and durability |
|  | Gas pathway biocompatibility   - Dry gas validation would include: Testing for volatile organic compounds and particulate matter sampling |
|  | Environmental controls to reduce transmission (e.g., negative pressure) |
|  | Leak tests on finished product |
|  | Design for use of disconnect alarms that are on multiple circuit paths |
|  | Compliance with guidelines regarding standard for ventilator circuitry ISO 5356-1 Third edition 2004-05-15 Anaesthetic and respiratory equipment - Conical connectors: Part 1: Cones and sockets |
|  | ISO 5366 First edition 2016-10-01 Anaesthetic and respiratory equipment - Tracheostomy tubes and connectors |
|  | ISO 18190 First edition 2016-11-01 Anaesthetic and respiratory equipment - General requirements for airways and related equipment |
|  | ISO 18562-1 First Edition 2017-03: Biocompatibility Evaluation of Breathing Gas Pathways in Healthcare Applications - Part 1: Evaluation and Testing Within a Risk Management Process |
|  | ISO 18562-2 First Edition 2017-03: Biocompatibility Evaluation of Breathing Gas Pathways in Healthcare Applications - Part 2: Tests for Emissions of Particulate Matter |
|  | ISO 18562-3 First Edition 2017: Biocompatibility Evaluation of Breathing Gas Pathways in Healthcare Applications - Part 3: Tests for Emissions of Volatile Organic Compounds |
|  | Appropriate labeling providing instructions for use and cautionary statements regarding the device use and recommended monitoring activities |
